# Multi-oxirane additives as property modifiers for DGEBA-based structural electrolytes

**DOI:** 10.1039/d6ta02802k

**Published:** 2026-10-02

**Authors:** Rajat Sharma, Sang Nguyen, Odysseas Kakarelidis, Emile S. Greenhalgh, Milo S. P. Shaffer, Valeria Arrighi, Leide Cavalcanti, Natasha Shirshova

**Affiliations:** a Department of Engineering, Durham University South Road Durham DH1 3LE UK natasha.shirshova@durham.ac.uk; b Department of Aeronautics, Imperial College London South Kensington London SW7 2AZ UK; c Department of Materials, Imperial College London South Kensington London SW7 2AZ UK; d School of Engineering and Physical Sciences, Institute of Chemical Sciences, Heriot-Watt University Edinburgh EH14 4AS UK; e Science Technology Facilities Council, Isis Neutron and Muon Source Harwell Campus Oxfordshire OX11 0QX UK

## Abstract

Structural supercapacitors require electrolytes that can efficiently transport ions while bearing mechanical loads, known as structural electrolytes (SEs). Here, we report the addition of the multi-oxirane epoxies, reactive epoxy additives (REAs), as a strategy to tune the microstructure of an established bicontinuous SE, formed by reaction induced phase separation (RIPS). Specifically, trimethylolpropane triglycidyl ether (TGETMP), 4-(diglycidylamino)phenyl glycidyl ether (GEDGP), tris(4-hydroxyphenyl)methane triglycidyl ether (TGETHP), and tetraglycidyl-4,4′-methylenedianiline (TGEMDA) were introduced into an SE based on a blend of bisphenol A diglycidyl ether (DGEBA) and 1-ethyl-3-methylimidazolium bis(trifluoromethylsulfonyl) imide (EMIM-TFSI), (DGEBA_IL40). Additive concentrations were varied between 1 and 50 wt% and the SEs were characterized using scanning electron microscopy, impedance spectroscopy, three-point bending tests and dynamic mechanical thermal analysis. At 2.5 wt% addition, all REAs enhanced ionic conductivity, but reduced Young's modulus, relative to the unmodified SE based on DGEBA_IL40. TGETMP showed the most promising performance by increasing ionic conductivity by 90% and reducing Young's modulus only by 20%. Small-angle neutron scattering of the curing process of DGEBA_IL40 with the addition of 2.5 wt% TGETMP showed a delay in phase separation kinetics and an increase in the cluster correlation length from about 30 Å to 63 Å compared to the unmodified system. Carbon aerogel-modified carbon fibre (CF-CAG) structural electrodes were introduced into the SEs, to explore whether the bicontinuous microstructure would be retained in a multifunctional device. The unmodified SE (DGEBA_IL40) showed epoxy sheathing on CF-CAG with bead-like morphology around fibres and strut-like morphology in the bulk, showing that the presence of CF-CAG influenced local curing of the SE at the electrode/electrolyte interface. Encouragingly, the addition of 2.5 wt% TGETMP to DGEBA_IL40 reduced both epoxy sheathing and bead formation around CF-CAG.

## Abstract 2

The effects of multi-oxirane ring epoxy additives on the microstructure, mechanical properties, ionic conductivity, and interfacial morphology of DGEBA-based structural electrolytes were studied.

## Graphical abstract

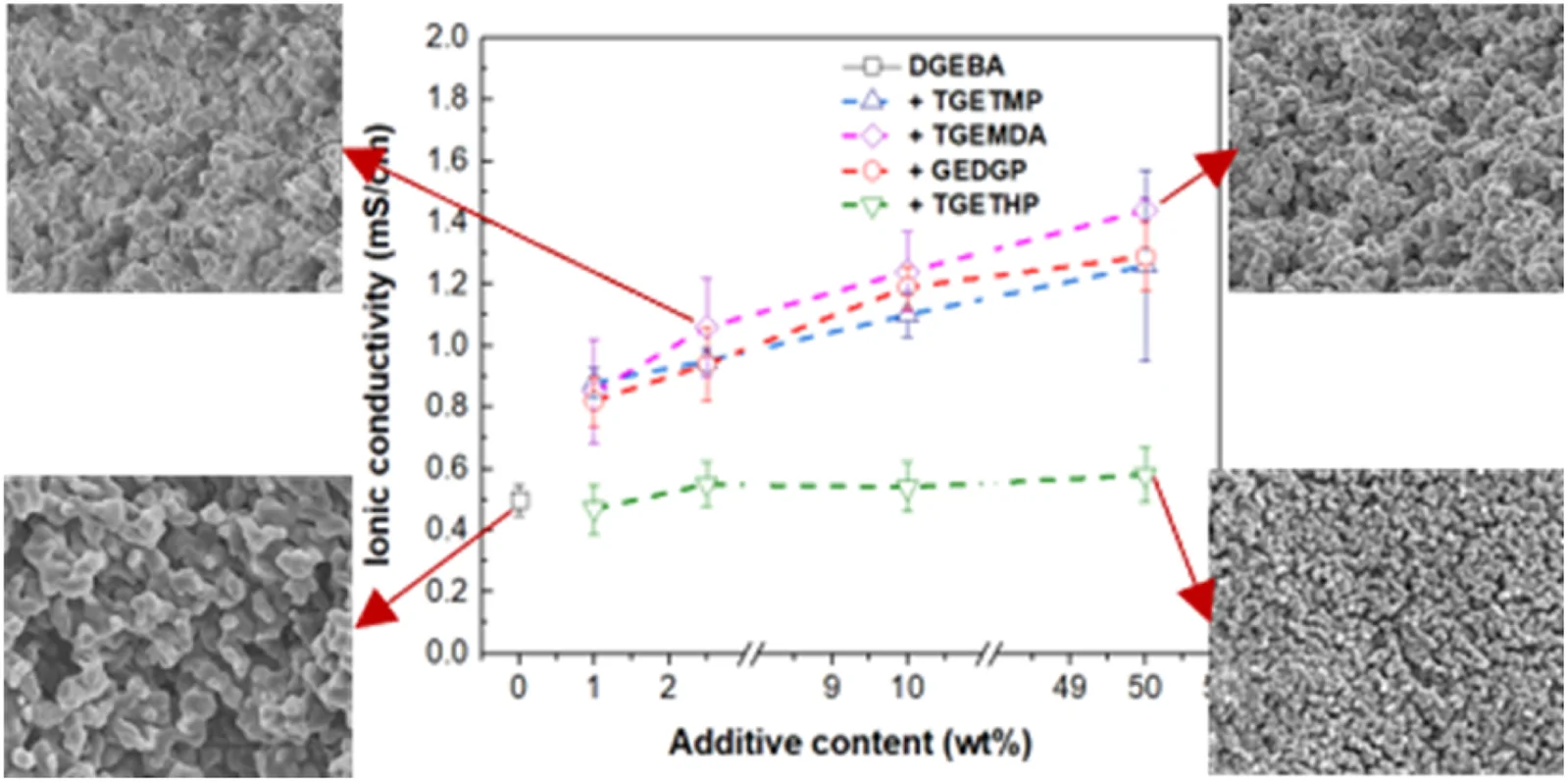

## 1 Introduction

Supercapacitors are energy storage devices, recognised for their high-power density, rapid charge–discharge capabilities, and long cycle life.^1,2^ While conventional supercapacitors focus on electrochemical performance,^2^ structural supercapacitors (SSCs) simultaneously carry meaningful mechanical loads.^3–6^ Composite SSCs rely on structural electrolyte (SE) matrices that must balance the conflicting requirements to conduct ions whilst retaining mechanical performance.^7^ Aspirational property targets for SEs are a Young's modulus above 1 GPa and an ionic conductivity greater than 1 mS cm^−1^.^4^ However, this multifunctionality presents a fundamental challenge,^8–11^ as ion transport benefits from polymer chain mobility, whereas a higher modulus requires a rigid network.

Liquid and gel electrolytes, despite their superior ionic transport properties, are unsuitable for structural applications due to their poor mechanical performance.^12,13^ Solid polymer electrolytes (SPEs), a blend of a polymer matrix and an alkali metal salt,^14–16^ can offer adequate stiffness at room temperature but generally exhibit significantly lower ionic conductivity under these conditions.^13^ To overcome this challenge, fillers, such as rigid fibres,^17,18^ nanofibers,^19,20^ metal–organic frameworks (MOFs),^21,22^ inorganic nanoparticles^23,24^ and others, have been incorporated into SPEs. Nonetheless, ionic conductivity (0.01 to 0.1 mS cm^−1^) still falls short of the levels needed for SEs in multifunctional energy storage devices.^16^

The most effective polymer-based SEs combine two separate continuously interpenetrating phases, one structurally rigid and one ionically conductive; these so-called bicontinuous electrolytes offer promising combinations of mechanical properties and room-temperature ionic conductivity.^4^ Bicontinuous SEs can be prepared *via* polymerisation-induced phase separation (PIPS), reaction-induced phase separation (RIPS) and by post-filling a pre-formed porous polymer matrix with a liquid electrolyte (LE) of choice.^11,26,31^ Similar to conventional SPEs and gel electrolytes,^13^ bicontinuous SEs show a trade-off between modulus and ionic conductivity, which can be tailored *via* changes in composition and the ratio of phases.^11,28,31^ Post-filled systems combine strong mechanical performance from the polymer skeleton, typically epoxy-based, obtained using RIPS or PIPS, with tuneable ionic conductivity from the chosen liquid electrolyte,^25–28^ but their multi-step synthesis complicates their scale-up and manufacturing of energy storage devices.

PIPS and RIPS systems start as a single-phase reaction solution containing monomer(s) and a LE. As the reaction progresses, two distinctive phases are formed, one responsible for mechanical performance and another for ionic conductivity (Fig. 1). For PIPS, multifunctional vinyl monomers (monomers containing more than one vinyl group, such as ethoxylated bisphenol A dimethacrylate, or their mixtures with other vinyl monomers) are combined with a variety of LEs.^29–33^ Most reported SEs prepared using PIPS are characterised by a wide range of properties and bicontinuous morphologies with polymer strut sizes below 100 nm.^30–34^ However, even for the most promising system, containing a poly(ethylene glycol) diacrylate (*M*_w_ = 1000 g mol^−1^) monomer with a bisphenol A ethoxylate dimethacrylate (*M*_w_ = 452.6 g mol^−1^) crosslinker as a structural phase and 1 M zinc(ii) bis(trifluoromethanesulphonyl)imide (Zn(TFSI)_2_) in succinonitrile as a LE, the best combination of Young's modulus (1.16 GPa) and ionic conductivity (0.32 mS cm^−1^) is still below the aspirational values.^31^

**Fig. 1:**
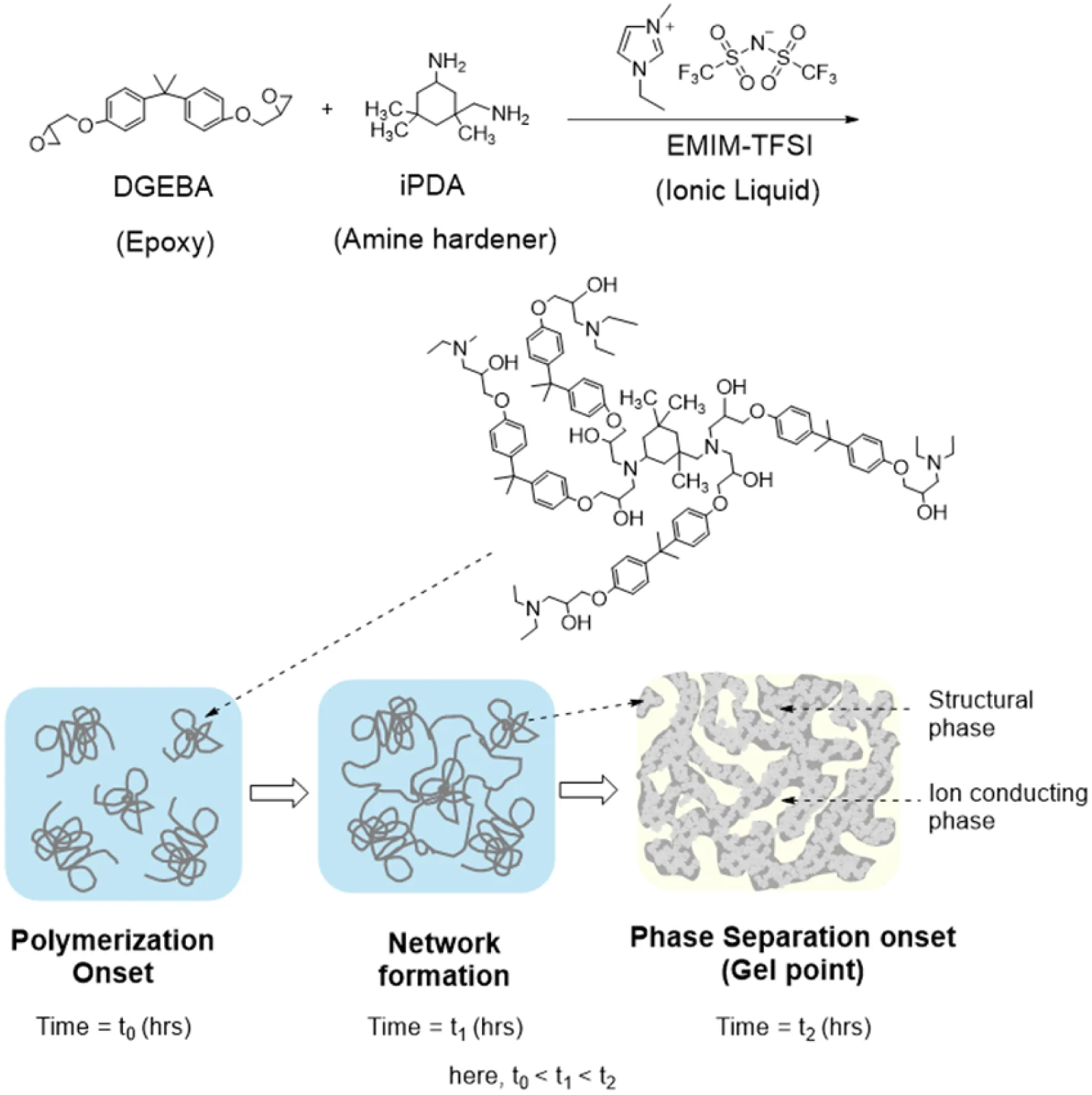
Illustration showing the network formation and phase separation of DGEBA polymerized with an amine hardener (iPDA) in an ionic liquid (EMIM-TFSI).

Most reported RIPS systems exploit epoxy resin(s), for their excellent mechanical properties and chemical stability, cured in the presence of a LE.^35,36^ The most studied epoxy resins are based on bisphenol A diglycidyl ether (DGEBA) systems.^11,38–43^ To introduce ionic conductivity, various LEs, including ionic liquids (ILs) and solutions of lithium salts in ILs or organic solvents, are added into DGEBA-based formulations.^38,42,43^ During the step-growth ring-opening polymerization of the DGEBA monomer in the presence of a LE, which is commonly initiated using an amine curing agent,^56^ the initially homogeneous DGEBA/LE mixture undergoes phase separation, resulting in a wide range of morphologies, from nodular structures^11,38^ to continuous struts,^11,43^ or a combination of both.^38,39^ The final structure reflects the competition between two processes, polymerization and phase separation,^57^ and can be tailored through varying the composition of the reaction mixture and/or curing conditions.^11,44^ Reported composition variations include the addition of homopolymers,^37^ block copolymers,^11^ inorganic fillers,^42^ and epoxy additives^38^ and adjusting the LE volume fraction.^11,38,42–44^

The addition of epoxy modifiers, especially those containing multi-oxirane rings, is of particular interest considering their potential for pre- or post-synthetic modification. Previous work on epoxy-based SPEs used multi-oxirane additives, *N*,*N*,*N*′,*N*′-tetraglycidyl-*m*-xylenediamine (Tetrad-X) and tetraglycidyl-4,4′-methylenedianiline (TGEMDA), to increase *T*_g_ of DGEBA/IL formulations, to an extent dependent on the structure of the epoxy additive; for Tetrad-X, *T*_g_ increased from 119 °C to 128 °C, whilst the addition of TGEMDA resulted in a more significant shift from 119 °C to 142 °C.^38^ The observed increase in *T*_g_ suggests reduced chain mobility in the epoxy network and, as a result, changes in the mechanical performance of the resulting polymers are expected but were not reported.

Despite these findings, the broader potential of the addition of multi-oxirane epoxies to the DGEBA/LE system remains unexplored. This omission is surprising as multi-oxiranes are frequently added to DGEBA-based structural resins (without a LE phase) to modify the network properties of these conventional thermosets.^45–48^ For instance, a blend of tetra-epoxy TGEMDA with DGEBA was used to increase the toughness of pure TGEMDA by reducing the crosslink density and, consequently, the brittleness of the cured epoxy system.^45^ Similarly, blends of tri-epoxy trimethylolpropane triglycidyl ether (TGETMP) with DGEBA showed increased fracture toughness at high TGETMP loadings (>40 wt%), although crosslinking density, flexural strength and elastic modulus decreased correspondingly.^47^ In the context of SEs, there is an interesting opportunity to modify the additional oxirane rings into cyclic carbonates that are commonly used as part of the electrolyte in lithium-ion batteries.^49^ This reactivity suggests that the introduction of multi-oxiranes may provide a potential route to simultaneously modify ionic conductivity and mechanical performance.

In epoxy/LE based SEs, curing kinetics governs the microstructure development,^11,65^ thereby controlling the resulting ionic conductivity and mechanical performance. However, in SSCs, SE curing occurs in the presence of electrodes and the separator^4^ which introduce additional interfacial interactions with the epoxy/LE phase, which may affect local cure kinetics and hence the resulting microstructure.^50^ SSC devices made with the CF fabric structural electrodes, plain weave glass fibre separators and an epoxy-based SE containing LiTFSI in EMIM-TFSI were previously reported to have a significantly modified microstructure compared to the neat cured SE.^10^ In addition to changes in the scale of microstructural features, the matrix-dominated mechanical properties were also reduced due to the formation of the EMIM-TFSI phase preferentially at the fibre surfaces.^10^ To produce effective multifunctional structural energy storage devices, it is essential to maintain a stable SE microstructure and strong fibre-matrix interfaces, upon incorporating the structural electrodes and a separator.

In this work, multi-oxirane epoxy resins are introduced as a novel strategy to tune the bicontinuous structure and multifunctional performance of the DGEBA/EMIM-TFSI based SE. The microstructure and properties, including mechanical performance, ionic conductivity and polymer strut size of the resulting SEs, are presented and discussed. In addition, the influence of reinforcing materials on SE morphology is examined using the widely adopted carbon aerogel (CAG) modified CF structural electrode system,^51^ addressing an important but underexplored aspect of practical SSC design.

## 2 Materials and methods

### 2.1 Materials

Bisphenol A diglycidyl ether (DGEBA), trimethylolpropane triglycidyl ether (TGETMP), 4-(diglycidylamino)phenyl glycidyl ether (GEDGP, ≥90%), tris(4-hydroxyphenyl)methane triglycidyl ether (TGETHP) and an isophoronediamine (iPDA, ≥99%) hardener were purchased from Sigma-Aldrich. Bis[4-(diglycidylamino)phenyl]methane (TGEMDA) was kindly provided by Hexcel. 1-ethyl-3-methylimidazolium bis(trifluoromethylsulfonyl) imide (EMIM-TFSI, ≥99%) was purchased from Iolitec Nanomaterials (Germany). All chemicals were used as received without further purification. The chemical structures of the compounds used in this work are presented in Fig. 2. The preparation of the reinforcement/electrode material, carbon aerogel reinforced CF (CF-CAG), is reported elsewhere.^51^ Spread tow carbon fibre (https://www.sciencedirect.com/topics/materials-science/carbon-fiber-tow) fabrics used to prepare CF-CAG were kindly provided by OXEON® AB (Sweden).

**Fig. 2:**
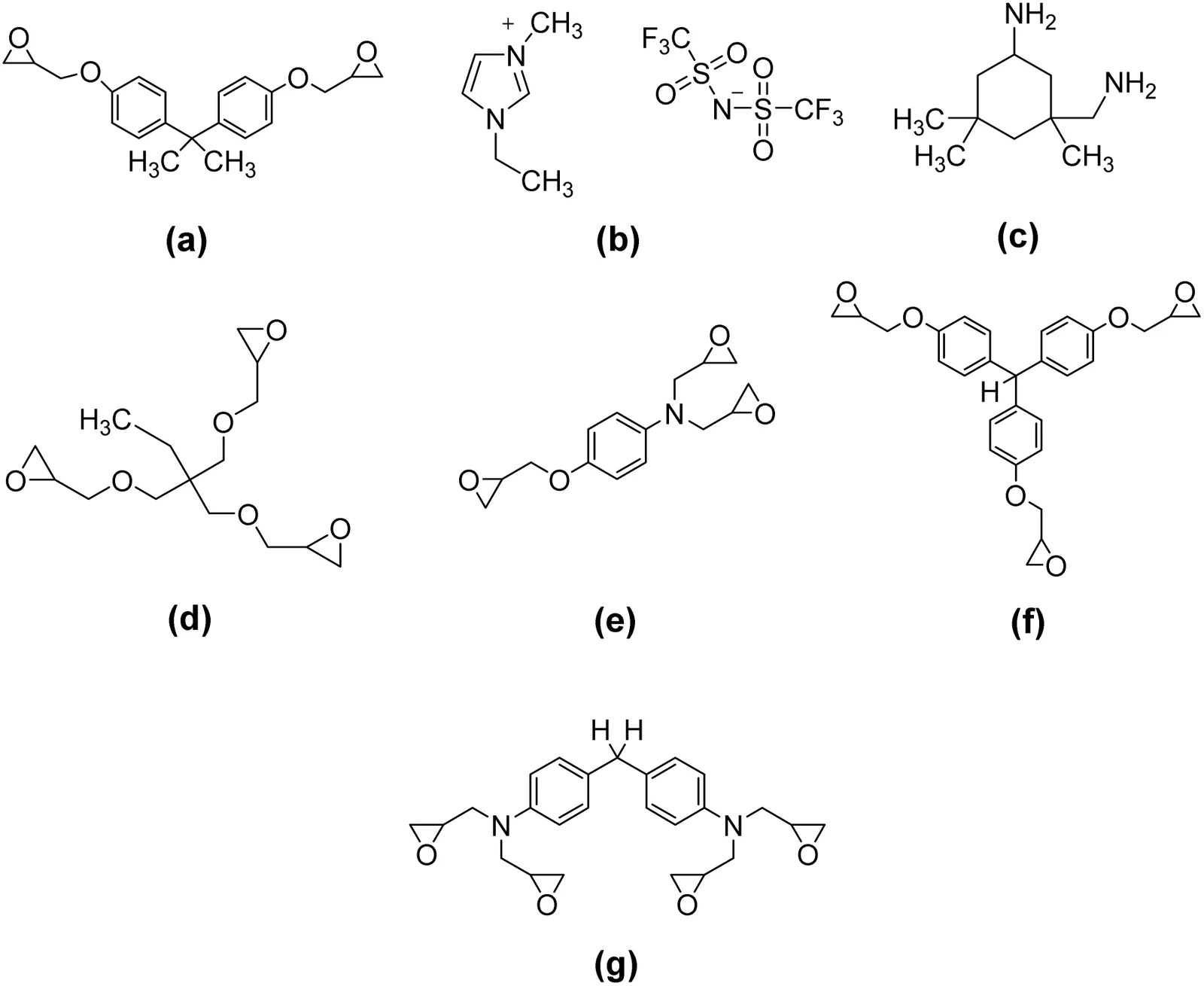
Chemical structures of the compounds used: (a) bisphenol A diglycidyl ether (DGEBA), (b) 1-ethyl-3-methylimidazolium bis(trifluoromethylsulfonyl) imide (EMIM-TFSI), (c) isophoronediamine (iPDA), (d) trimethylolpropane triglycidyl ether (TGETMP), (e) 4-(diglycidylamino)phenyl glycidyl ether (GEDGP), (f) tris(4-hydroxyphenyl)methane triglycidyl ether (TGETHP) and (g) tetraglycidyl-4,4′-methylenedianiline (TGEMDA).

### 2.2 Preparation of the structural electrolytes

All the DGEBA-based SE formulations studied (Table 1) were prepared following a procedure adapted from Wendong *et al.*^11^ Neat DGEBA was pre-heated at 50 °C until fully melted, held for 10–15 min to eliminate residual crystallinity, and then cooled to room temperature (RT). For the formulations containing reactive epoxy additives (TGETMP, TGETHP, TGEMDA, and GEDGP), the additive was first incorporated into DGEBA at RT using a planetary centrifugal mixer (Thinky ARM310) at 2000 rpm for 1 min. The loadings of the reactive additives were calculated as weight percentage (wt%) relative to DGEBA only. EMIM-TFSI (IL) was then added to uncured DGEBA (with and without reactive additives) at RT to achieve 40 vol% of the total formulation. The mixture was homogenized using a planetary centrifugal mixer at 2000 rpm for 1 min. The total epoxy content, including contributions from both DGEBA and any additives, was used to calculate the required amount of isophorone diamine (iPDA) to maintain the desired stoichiometric ratio: 2 : 1 epoxy : iPDA for di-epoxides, 1.5 : 1 for tri-epoxides, and 1 : 1 for tetra-epoxides. The calculated amount of iPDA was then added to the formulation and mixed again at 2000 rpm for 1 min, forming a solution.

**Table 1:** Composition of the formulations studied^a^

Sample	DGEBA (g)	iPDA (g)	EMIM-TFSI (g)	REA (g)
DGEBA pure	10.0	2.5	—	—
DGEBA_IL40	10.0	2.6	11.6	—
TGETMP_2.5%_IL40	10.0	2.6	11.8	0.25
TGETHP_2.5%_IL40	10.0	2.6	11.8	0.25
TGEMDA_2.5%_IL40	10.0	2.6	11.8	0.25
GEDGP_2.5%_IL40	10.0	2.6	11.8	0.25
TGETHP_50%_IL40	10.0	3.7	17.1	5.00

a DGEBA pure is DGEBA/iPDA cured without EMIM-TFSI (IL); DGEBA_IL40 is DGEBA/iPDA cured with 40 vol% IL; TGETMP/TGETHP/TGEMDA/GEDGP_wt%_IL40 is DGEBA/iPDA with different wt% reactive epoxy additives (REAs) cured with 40 vol% IL. The prepared solutions were transferred into three different moulds (Fig. S1) depending on the intended analysis of the final polymeric material: (i) for mechanical analysis (3-point bending) the sample was cured vertically in a flat glass mould (50 mm × 80 mm × 2 mm) coated with a CR1 Easy-Lease chemical release agent (EasyComposites), (ii) for ionic conductivity measurements in 5 mL syringes (diameter = 9.6 ± 0.3 mm), and (iii) to assess the effect of the reinforcement/electrode material on the microstructure formation during curing in 20 mL polypropylene beakers containing two vertically positioned layers of structural electrode CF-CAG (∼30 mg).

All samples were cured in a fan-assisted oven (Lefton LF15) equipped with a Eurotherm (3216P1) temperature controller, applying the following curing cycle: (1) dwell at RT (25 °C) for 18 h, (2) a ramp of 2 °C min^−1^ to 60 °C, (3) dwell at 60 °C for 1 h, (4) a ramp of 2 °C min^−1^ to 80 °C, (5) dwell at 80 °C for 2 h, (6) a ramp of 2 °C min^−1^ to 120 °C, (7) dwell at 120 °C for 2 h, (8) cooling down to RT.^11^ To ensure that sample mould geometry had minimal effect on SE curing, the properties of DGEBA containing 40 vol% IL-based SE cured in a flat glass mould and a syringe were compared (Fig. S2).

### 2.3 Characterization of the structural electrolyte

The ionic conductivity of the SEs was determined for as-synthesised samples using electrochemical impedance spectroscopy (EIS) in a two-electrode configuration, where SE discs were sandwiched between two stainless steel electrodes. Measurements were conducted using a VersaSTAT 3-450 potentiostat (AMETEK) over a frequency range of 250 kHz to 0.1 Hz with a 5 mV AC amplitude at room temperature (20 ± 2 °C). Cylindrical specimens cast in syringes were sectioned into two disc-shaped samples (9.5 ± 0.1 mm diameter), taking one each from the top and bottom parts to assess homogeneity. The discs were initially polished with 80-grit sandpaper and subsequently refined with 120-grit sandpaper to achieve flat and parallel surfaces. The thickness of each disc was measured (2.0 ± 0.1 mm) using a digital Vernier calliper (Mitutoyo, ±0.01 mm). To minimise interfacial resistance and compensate for potential loss of EMIM-TFSI during surface preparation, a drop of EMIM-TFSI (∼0.1 mL) was applied to both disc faces and left for 10 min each side to settle before measurements. The disc sides were wiped to remove any excess EMIM-TFSI, ensuring that measurements were taken exclusively through the thickness of the disc. For each formulation, at least four measurements were taken, and the ionic conductivity (*σ*) was calculated as1
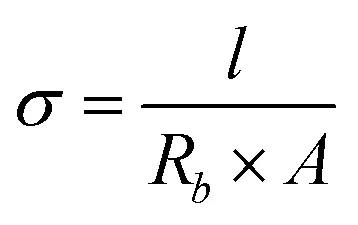
where *σ* is the ionic conductivity (S cm^−1^), *l* is the thickness of the disc (cm), *A* is the contact area (cm^2^), and *R*_b_ (Ω) is the bulk resistance of the SE disc, obtained from the intercept of the high frequency region of the Nyquist plot with the real part (*x*-axis).^11^

The mechanical performance of the cured formulations was evaluated at room temperature (20 ± 2 °C) on as-synthesised samples using three-point bending (3PB).^11^ The setup uses three polished steel cylinders with a 19.5 mm span and a loading roller with a 4 mm diameter. Rectangular specimens (24 mm × 5 ± 0.2 mm × 2 ± 0.1 mm) were cut from cured SE plaques. Tests were conducted until 10% sample deformation at a constant displacement rate of 0.5 mm min^−1^ using an LR5KPlus universal testing machine (Lloyd Instruments/Ametek) equipped with a 500 N load cell.^11^ For each formulation, a minimum of five specimens were tested, and the mean ± standard deviation were reported. The Young's modulus (*E*_b_) was calculated as2
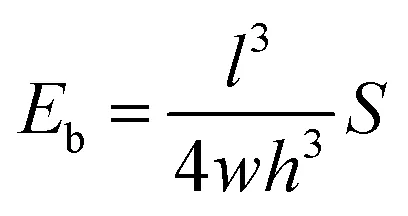
where *E*_b_ is the Young's modulus (MPa), *l* is the span length (19.5 mm), *w* is the specimen width (mm), *h* is the thickness of the specimen (mm), and *S* is the slope (N mm^−1^) of the initial linear region of the force–displacement curve.

Dynamic mechanical analysis was performed using a Netzsch DMA 303 Eplexor in three-point bending mode (span length: 20 mm) at a frequency of 1 Hz. The temperature was ramped from 25 °C to 200 °C at a rate of 2 °C min^−1^. The measurement parameters were as follows: an amplitude of 40 µm, a force factor of 1.2, a dynamic force limit of 15 N, and a contact force of 0.1 N. Three repeats were performed per formulation, and the glass transition temperature (*T*_g_) was determined as the maximum of the tan  *δ* curve. With an assumption that the material would behave as an ideal elastomer in the rubbery regime, the average molecular weight between cross-links (*M*_c_, g mol^−1^) was calculated as3
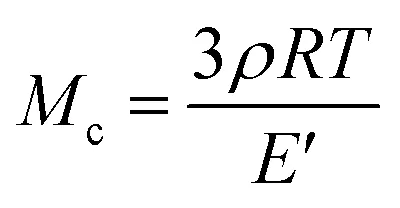
where *E*′ (MPa) is the storage modulus at *T*_g_ + 40 °C, *R* is the universal gas constant (8.314 m^3^ Pa K^−1^ mol^−1^), *ρ* is the density at 25 °C and *T* is the temperature (*K*) at which *E*′ is obtained.^52^

The apparent crosslink density (*ν*_e_, mol cm^−3^), defined as the number of elastically effective chains per unit volume, was calculated as^55^4
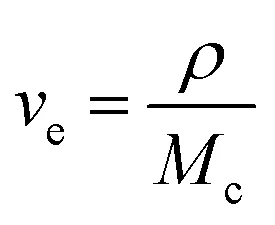


The density of the cured samples was determined using an AccuPyc 1330 pycnometer, which measures sample volume by monitoring the pressure change of nitrogen gas in a calibrated chamber. One sample per formulation was tested at 25 °C.

Small-angle neutron scattering (SANS) measurements were conducted on the SANS 2D and ZOOM instruments at the STFC ISIS Pulsed Neutron Source (Rutherford Appleton Laboratory, Didcot, UK). All experiments were performed *in situ* during room-temperature curing. Background measurements utilized Hellma liquid cells, while the curing kinetics were investigated using modified Durham cells featuring a 1 mm path length, 10 mm inner diameter, and quartz windows. The *Q* range (*Q* = (4π/*λ*)sin(*θ*/2), where *θ* is the scattering angle and *λ* the neutron wavelength), explored on ZOOM varied from 0.0025 to 0.5 Å^−1^, whereas on SANS 2D the *Q*-range was 0.0015 to 0.5 Å^−1^. All scattering data were normalised for sample transmission and the incident wavelength distribution and corrected for instrumental and sample backgrounds using an empty cell. The scattered intensity (*I*(*Q*)) was converted to normalized differential scattering cross-section per unit volume, d*Σ*(*Q*)/d*Ω*, expressed in units of cm^−1^ using a blend of hydrogenated and deuterated polystyrene standards. Background samples included (1) DGEBA:d-IL 60 : 40 vol%; (2) deuterated IL (d-IL); (3) hydrogenated IL (h-IL); (4) iPDA:d-IL; (5) TGETMP; (6) an empty cell. These samples showed no visible structural features and only background scattering was measured. Analysis was done using SASView 6.0.1.^53^

To examine the morphology of the cured SE, the ionic liquid was extracted from the cured formulations. Disc-shaped specimens (2.0 ± 0.1 mm thick) were cut from the cured cylinders, weighed, and transferred into 20 mL glass vials. Methanol (5–10 mL) was added as the extraction solvent. Samples were subjected to five successive 15-minute extraction cycles, followed by two overnight extractions. After extraction, the specimens were dried overnight in an oven at 60 °C until a constant weight. For SE samples with CF-CAG, 10 mL of methanol was added directly to the polypropylene cup containing the SE with CF-CAG, and the same extraction procedure was applied. After drying, the sample was manually delaminated, and SEM specimens were prepared and imaged from the separated CF-CAG layers, following the procedure outlined below.

Morphological characterization was conducted using an FEI Helios Nanolab 600 Scanning Electron Microscope (SEM) at accelerating voltages of 5–10 kV.^11^ Aluminium pin stubs (9.5 mm diameter, Agar Scientific) were first covered with conductive carbon stickers to secure copper tape. Freshly fractured samples were then mounted onto the copper tape and sputter-coated with a *ca.* 15 nm platinum layer using a Cressington 328 system, with thickness monitored using a Cressington MTM10. Morphological features were quantified using ImageJ software.^54^

## 3 Results and discussion

Here, multi-oxirane ring epoxies, or reactive epoxy additives (REAs), TGEMDA, GEDGP, TGETMP, and TGETHP, were introduced at concentrations ranging from 1 to 50 wt% into DGEBA/iPDA formulations containing 40 vol% of EMIM-TFSI to establish their effect on the microstructure and properties of the resulting polymers. The volume fraction of the EMIM-TFSI, 40 vol%, was chosen as a baseline to provide a clear comparison to the previous work.^11^ All studied formulations remained macroscopically homogeneous during curing, with no evidence of gross/macrophase separation. The lack of gross separation was established visually and by determining the ionic conductivity for the top and the bottom of the cylindrical specimens (Fig. S3). If present, gross phase separation would result in samples with polymer-rich upper and ionic liquid-rich lower parts of the sample, due to the difference in densities between the polymer and ionic liquid, leading to very different ionic conductivity values.^11^

### 3.1 Effect of reactive epoxy additives on the ionic conductivity and microstructural properties of the structural electrolyte

The incorporation of REAs into DGEBA–EMIM-TFSI formulation (DGEBA_IL40) had a significant impact on ionic conductivity values and the microstructure of the resulting polymers. The SEM images show the typical bicontinuous morphology reported previously for DGEBA_EMIM-TFSI systems,^11^ comprising interconnected polymer struts and inter-strut spacing/regions (Fig. 3(a)–(f) and S4). For all studied REAs, an addition of 1 wt% led to a decrease in polymer strut size with minimal change in inter-strut spacing when compared to DGEBA_IL40 (Fig. 3(a), S4 and 4(a), (b)).

**Fig. 3:**
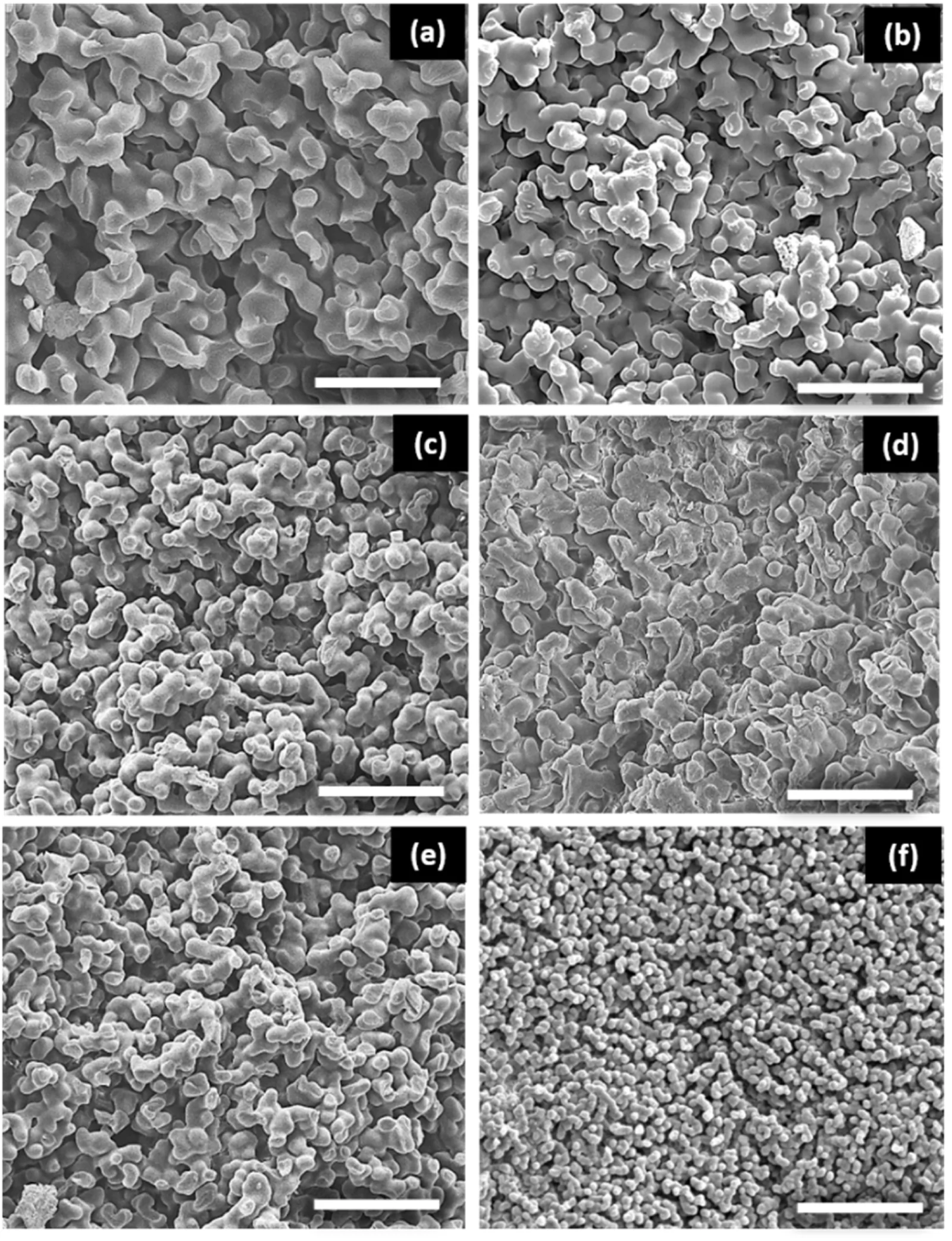
Scanning electron micrographs of DGEBA-based structural electrolytes with 40 vol% EMIM-TFSI (IL): (a) DGEBA_IL40, containing different reactive additives: (b) TGETMP_2.5%_IL40, (c) GEDGP_2.5%_IL40, (d) TGEMDA_2.5%_IL40, (e) TGETHP_2.5%_IL40 and (f) TGETHP_50%_IL40. Scale bar = 10 µm.

**Fig. 4:**
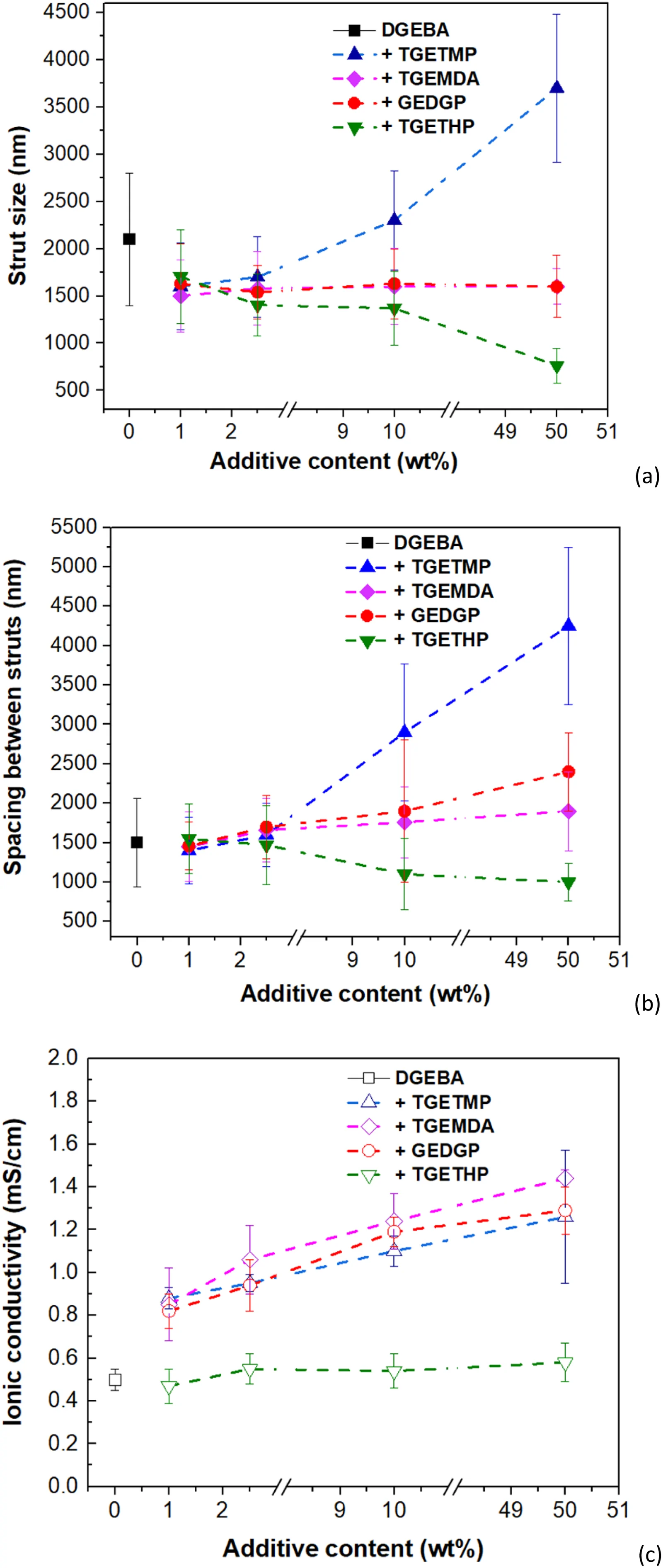
Effect of reactive additive concentration on (a) polymer strut size, (b) spacing between polymer struts, and (c) ionic conductivity (additive wt% is relative to 100 wt% DGEBA).

Increasing the additive content led to varied effects, depending on the REA used. For TGETMP, a branched, aliphatic epoxy with three oxirane rings (Fig. 2(d)), both strut size and inter-strut spacing increased progressively with concentration from 1 wt% to 50 wt% (Fig. 3(b), S4 and 4(a), (b)), leading to a less compact morphology and improved ionic conductivity (Fig. 4(c)). TGETHP, a sterically hindered aromatic epoxy with three oxirane rings (Fig. 2(f)), exhibited an opposite trend, with a decrease in ionic conductivity accompanied by reductions in structural feature sizes (Fig. 3(e), S4 and 4(a), (b)). At 50 wt% TGETHP, the resulting morphology was markedly finer (Fig. 3(f)), with polymer strut dimensions reduced by ∼60% compared with DGEBA_IL40 (Fig. 3(a)), while maintaining comparable ionic conductivity (Fig. 4(c)). In contrast to TGETMP and TGETHP, addition of TGEMDA (Fig. 2(g)) and GEDGP (Fig. 2(e)) in the studied concentration range did not lead to significant changes in strut sizes, but did increase inter-strut spacing in the resulting polymers (Fig. 3(c), (d) and Fig. S4). The observed conductivity increases for TGEMDA and GEDGP (Fig. 4(c)) likely arise from the combined effect of the increased inter-strut spacing and potential synergistic effects of polar functionalities (oxygen, nitrogen and hydroxyl groups) that facilitate ion mobility.^58^

In SEs containing ionic liquids such as EMIM-TFSI, ion mobility is primarily governed by electrostatic and ion–polymer interactions. The [EMIM]^+^ is known to exhibit weaker electrostatic interactions with [TFSI]^−^ owing to their size difference in comparison to other ionic liquids such as EMIM-OAc.^59^ Because of the difference in ion size, the [TFSI]^−^ diffuses more slowly than [EMIM]^+^; as a result, overall conductivity in EMIM-TFSI is determined by the faster moving [EMIM]^+^.^59^ Apart from the [EMIM]^+^–[TFSI]^−^ ion pair, formation of other ion pairs, such as [EMIM]^+^–[EMIM]^+^ and [TFSI]^−^–[TFSI]^−^, has also been reported and been proposed to restrict local ion mobility.^60^ Atomistic simulations have shown that the relatively weak, long-range [EMIM]^+^–[EMIM]^+^ and [TFSI]^−^–[TFSI]^−^ interactions contribute less to the restrictions of ionic mobility locally than [EMIM]^+^–[TFSI]^−^ which is stabilized by strong H-bonding and electrostatic interactions.^60^ In a system containing a polymer and electrolyte, addition of polar functionalities, such as nitrogen, oxygen, carbonyl or hydroxyl groups, in the polymer backbone has been shown to increase the polymer dielectric constant and reduce ionic aggregation of the electrolyte, leading to greater uncorrelated ionic motion and improved ionic conductivity.^61^ However, the relationship between polymer polarity and ionic conductivity is not linear. Increasing the polymer polarity beyond a certain value, which is polymer dependent, results in an increase in polymer–polymer and polymer–ion interactions, slowing polymer segmental dynamics and thereby reducing electrolyte conductivity.^61^

In crosslinked systems such as DGEBA/iPDA, although polymer segmental mobility is restricted and coordination sites are limited, accessible polar groups, mainly nitrogen, oxygen and hydroxyl groups in the polymer backbone (Fig. 5), could enhance [EMIM]^+^–[TFSI]^−^ ion pair dissociation.

**Fig. 5:**
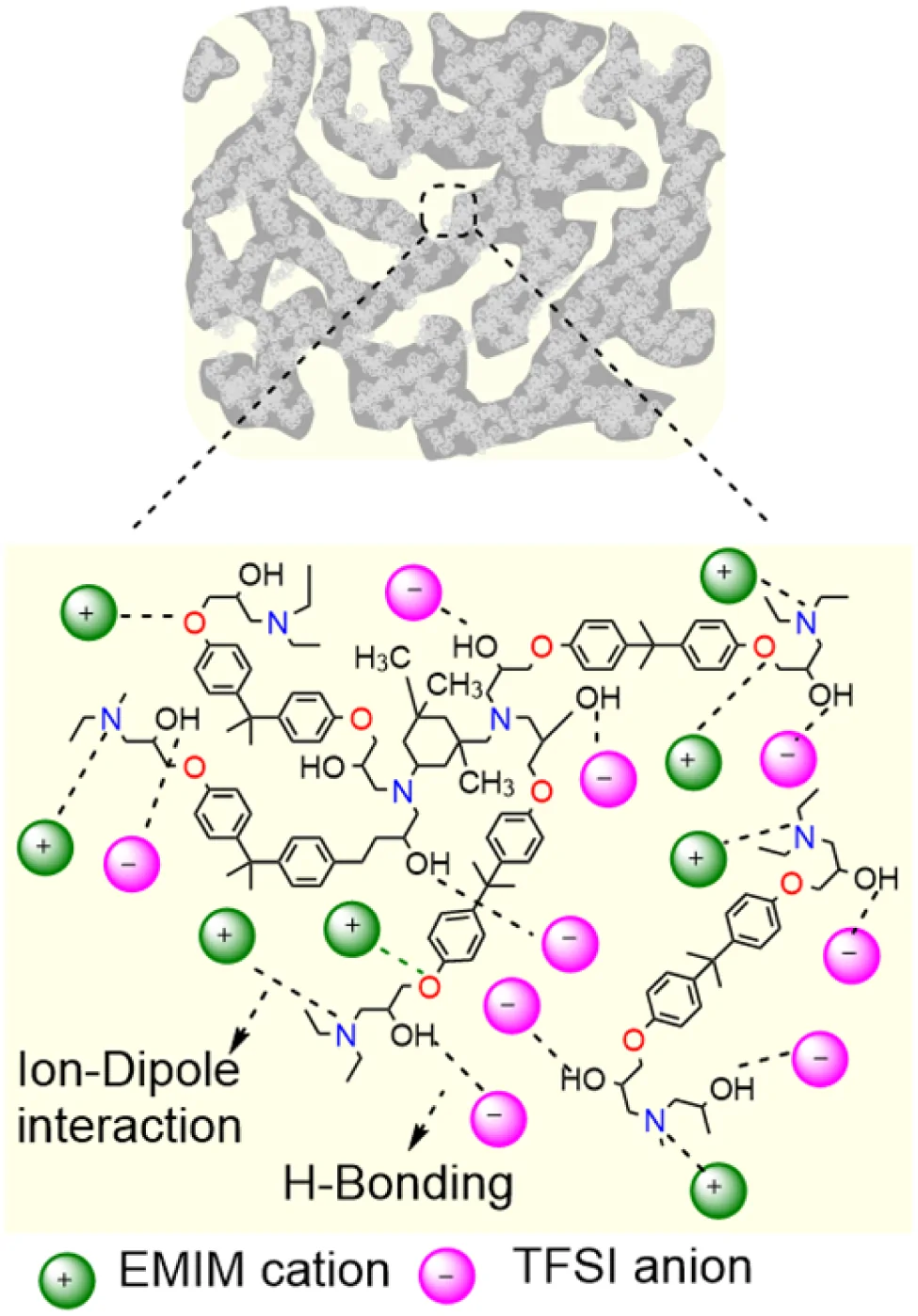
Schematic representation of the proposed ionic interactions in DGEBA/EMIM-TFSI-based polymer electrolytes, highlighting the role of non-covalent interactions.

Apart from epoxy/amine reactions, it is also expected that [EMIM]^+^ can take part in the ring opening of the oxirane rings,^62,63^ generating extra hydroxyl-rich poly(ether) segments,^63^ which could further increase the local polarity. However, since the curing reaction does not go above 120 °C, the contribution from [EMIM]^+^ reactivity is probably small compared to the conventional iPDA hardener.^64^ Nevertheless, if a proportion of [EMIM]^+^ reacts with the oxirane ring of the epoxy, the polarity of the resulting polymer chains would increase, leading to interactions between the DGEBA/iPDA polymer network and EMIM-TFSI mainly *via* hydrogen bonding and ion–dipole interactions (Fig. 5). These interactions could lead to disruption of the ionic pairs thereby increasing the fraction of free ions and enhancing charge transport. In addition, the incorporation of REAs also increases the number of polar functionalities within the DGEBA/iPDA network, which may further promote ion dissociation and contribute to the observed increase in ionic conductivity (Fig. 4(c)).

### 3.2 Microstructure formation observation using SANS

The effect of adding 2.5 wt% TGETMP to the DGEBA_IL40 formulation on microstructure development was investigated using small-angle neutron scattering (SANS), in comparison to the unmodified resin (DGEBA_40IL formulation) (Fig. 6). The differences in the *Q*-range between the two samples arise from the use of different instruments: SANS 2D, used for the 2.5 wt% sample, offers a broader *Q*-range compared to ZOOM (0.0015 to 0.5 Å^−1^). A detailed analysis of the DGEBA_IL40 curing in 50 : 50 wt% dIL : hIL can be found elsewhere.^65^

**Fig. 6:**
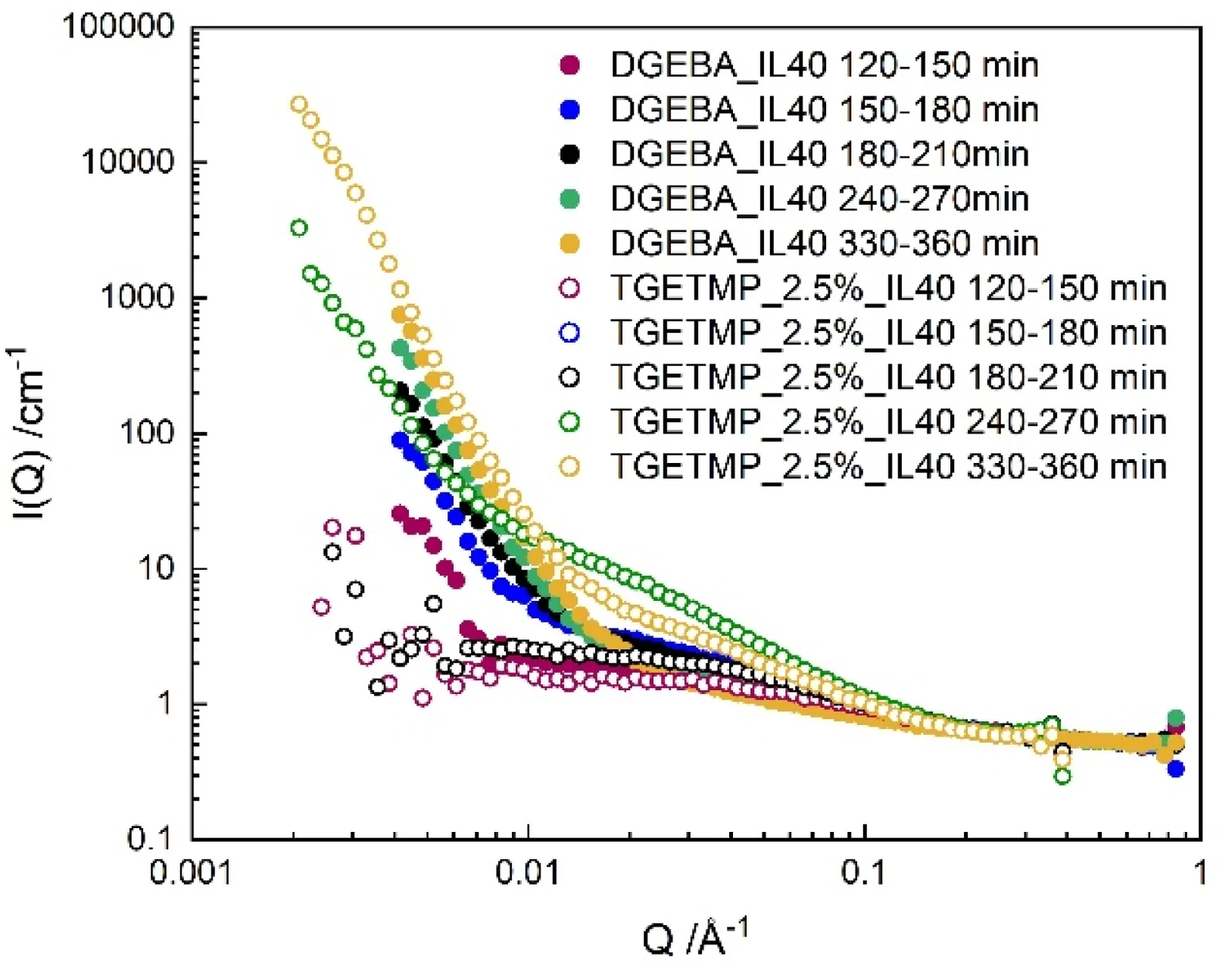
Comparison of SANS data at selected curing times for EP50dIL (DGEBA_IL40) (filled circles) and DGEBA containing 2.5 wt% TGETMP in dIL (TGETMP_2.5%_IL40, empty circles) as a function of curing time. The sample consists of epoxy and iPDA in a mixture of d-IL : h-IL (50 : 50 wt%, *i.e.* 50dIL for DGEBA) and dIL for the sample with 2.5 wt% TGETMP. EP : iPDA = 4 : 1 wt/wt; [IL] = 40 vol%.

The SANS data show that, for curing times up to 90 min, at room temperature, both formulations remain single-phase, which is indicative of a homogeneous solution, as indicated by the flat curves especially at *Q* > 0.005. These early time scattering curves are not shown, because they are flat and statistically indistinguishable. At longer curing times, the SANS traces exhibit a pronounced upturn at *Q* values below 0.01 Å^−1^. At the same time, a broad shoulder appears in the intermediate *Q* range (0.01 to 0.5 Å^−1^) and a plateau at *Q* > 0.5 Å^−1^, due to incoherent scattering. Similar features were observed for both formulations. To extract quantitative information from the SANS data, three different models were used.^56^

As previously reported, to analyse the curing kinetics of DGEBA in 50dIL (solution of 50 : 50 wt% deuterated (dIL) and hydrogenated (hIL) EMIM-TFSI) two different models were required.^65^ For the early stages of the curing process, SANS data were fitted using the relationship5
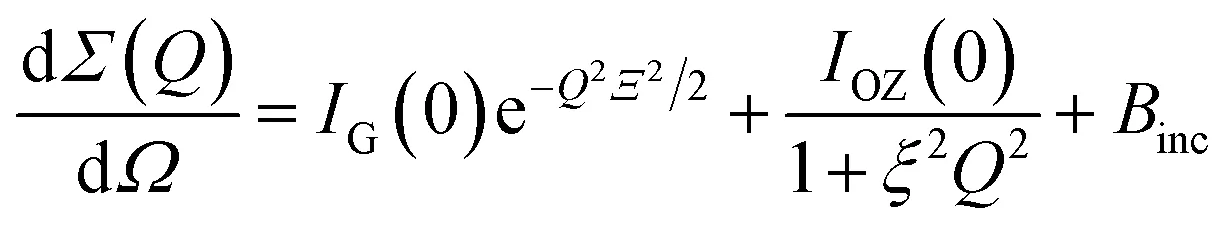


The first term provides an expression of the large-scale structures observed at low *Q* with *Ξ* representing the length scale of the solid-like (static) correlations. The second term is the Ornsteine–Zernike (OZ) equation, often used to describe the scattering of polymer solutions with *ξ* corresponding to a correlation length. The parameters *I*_G_(0) and *I*_OZ_(0) quantify the scattering intensity at *Q* = 0 of the two different contributions. *B*_inc_ is a *Q* independent background, largely due to incoherent scattering.

For curves recorded after 180 min of curing, a model proposed by Hammouda *et al.* was used to fit the data.^66,67^6
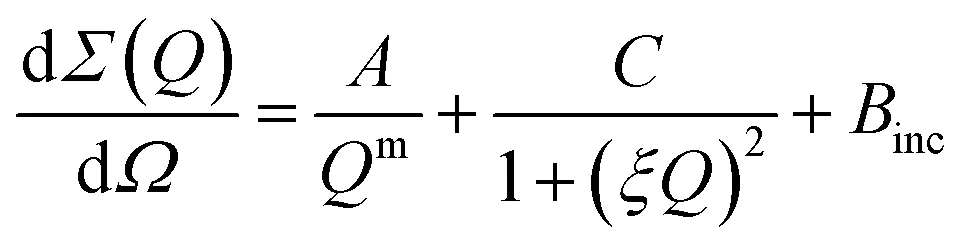


The first term in eqn (6) represents Porod scattering: *m* is the Porod exponent and *A* is a scaling factor. The second term is a Lorentzian function describing, in this case, scattering from thermal fluctuations.

For the formulation containing 2.5 wt% TGETMP, a single model (the two Lorentzian model in SAS View) successfully described the SANS data:7
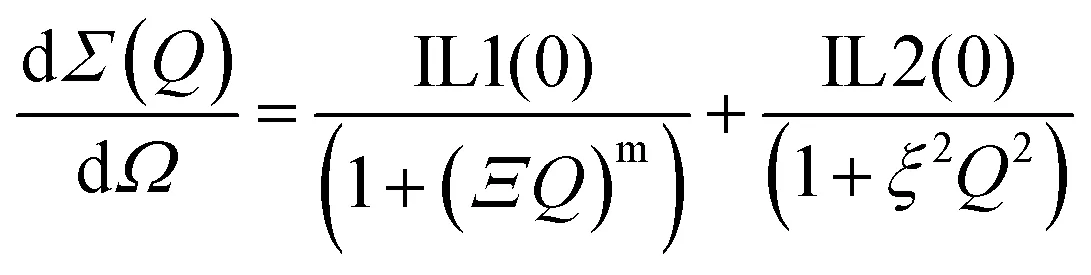
where IL1(0) is Lorentzian scale factor 1, IL2(0) is Lorentzian scale factor 2.

Comparisons between the correlation length fitting parameters, *ξ* and *Ξ*, show that (Fig. 7(a)) the addition of 2.5 wt% TGETMP delayed the formation of small phase segregated clusters; not only is the *ξ* maximum shifted to longer times but significantly larger clusters (63 Å) also form in comparison to the formulation without TGETMP (30 Å). The larger correlation length, *Ξ*, does not change until *ca.* 200 min, at which point values increase rapidly (Fig. 7(b)). These results suggest that for the formulation containing 2.5 wt% TGETMP, phase separation is delayed compared to the DGEBA_IL40 system. The lack of data points for the DGEBA system after 200 min of curing is a result of using the Porod equation to model the low *Q* scattering. The first term in eqn (6) describes large scale phase separation but does not provide values of correlation length.

**Fig. 7:**
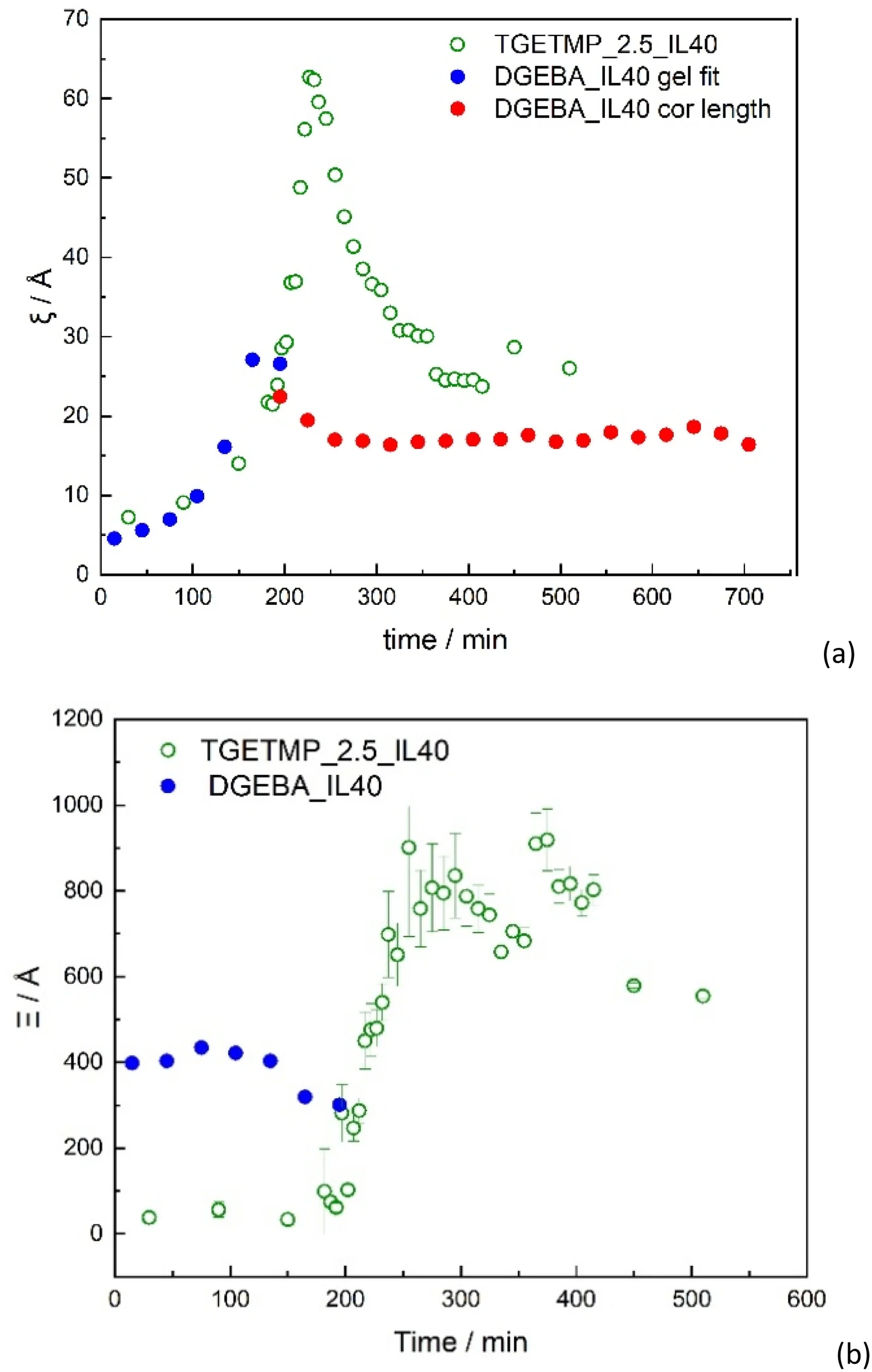
Fitting parameters obtained from the SANS data of DGEBA_IL40 (50dIL : 50hIL) and TGETMP_2.5%_IL40 (dIL; green empty circles): (a) short correlation length, *ξ*, as obtained from fits using eqn (5) (blue symbols), eqn (6) (red symbols) and eqn (7) (green empty circles); and (b) long correlation length, *Ξ*.

### 3.3 Effect of the reactive additive on the mechanical properties of the structural electrolyte

To further evaluate how REAs influence the properties of the crosslinked structure, selected formulations were characterised using DMTA and subjected to 3PB tests (representative curves in Fig. S5). Formulations containing 2.5 wt% of each REA were chosen because they produced minimum microstructural changes in comparison to DGEBA_IL40 (Fig. 3(a)–(e)), allowing a direct comparison of the effect of the additives on network properties without interference from morphological changes. In addition, a formulation containing 50 wt% TGETHP was selected as it showed significantly reduced polymer strut dimensions (Fig. 3(f)) combined with ionic conductivity comparable to that of DGEBA_IL40.

The formulations containing 2.5 wt% REA showed reduced Young's moduli relative to the DGEBA_IL40 baseline, to an extent (20–40%) depending on the specific modifier used (Table 2, Fig. 8). The trifunctional TGETMP showed the smallest reduction in Young's modulus, whereas tetrafunctional TGEMDA resulted in a 40% reduction relative to DGEBA_IL40. Similar behaviour has been reported in DGEBA blends with REAs, where a concurrent decrease in *T*_g_ and flexural modulus was associated with reduction in the cross-link density.^45,47^ REAs can increase network heterogeneity in the DGEBA-based epoxy network, as differences in reactivity can lead to the formation of additive-rich and matrix-rich domains, resulting in spatial variations in cross-link density, which in turn influence mechanical properties.^68,69^

**Table 2:** Effect of the composition on the properties and morphological characteristics of pure DGEBA and DGEBA based SEs with 40 vol% EMIM-TFSI. For feature size, ± denotes standard deviation, *n* ≥ 50

Sample code	Ionic conductivity (mS cm^−1^)	Young's modulus (GPa)	Feature sizes (µm)	Storage modulus *E*′ at 25 °C (GPa)	*T*_g_ (°C)	Molecular weight between cross-links, *M*_c_ (g mol^−1^)
Pure DGEBA	—	3.30 ± 0.38	—	2.80	160	500
DGEBA_IL40	0.50 ± 0.05	1.39 ± 0.32	2.1 ± 0.7	1.20	139	1840
TGETMP_2.5%_IL40	0.95 ± 0.04	1.09 ± 0.11	1.7 ± 0.4	1.08	138	1830
TGETHP_2.5%_IL40	0.55 ± 0.07	0.84 ± 0.03	1.4 ± 0.3	0.85	140	1870
GEDGP_2.5%_IL40	0.94 ± 0.12	0.83 ± 0.08	1.5 ± 0.3	0.75	140	1860
TGEMDA_2.5%_IL40	1.06 ± 0.16	0.80 ± 0.06	1.6 ± 0.4	0.90	139	1850
TGETHP_50%_IL40	0.58 ± 0.09	0.40 ± 0.04	0.8 ± 0.2	0.35	150/180^a^	3000/3200

a Two *T*_g_ peaks were observed for TGETHP_50%.

**Fig. 8:**
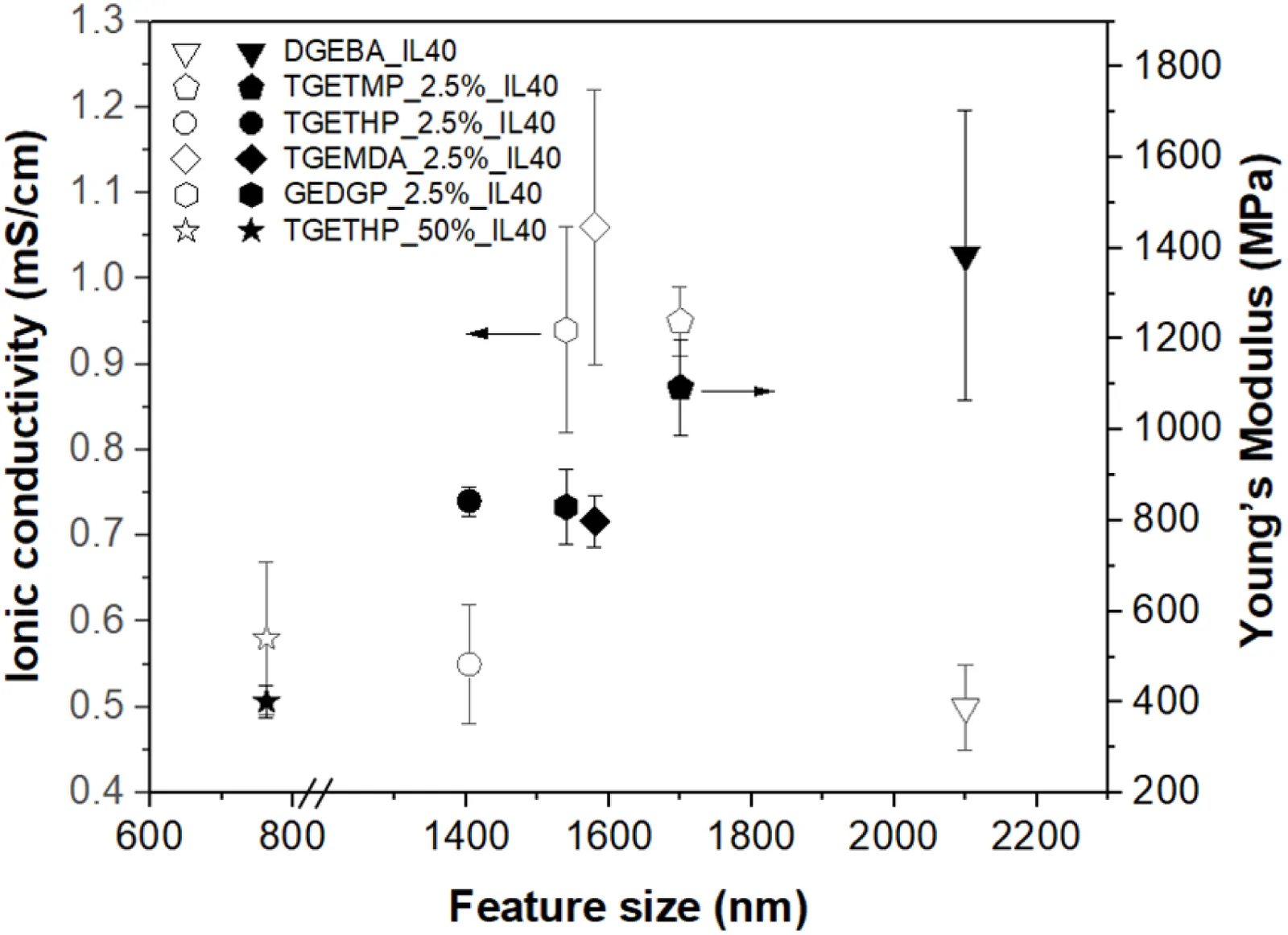
Effect of the reactive additives on the properties of the DGEBA-based structural electrolytes with 40 vol% EMIM-TFSI. Here, solid symbols are for Young's modulus and hollow symbols are for ionic conductivity.

Storage modulus values obtained from DMTA showed good agreement with the 3PB results (Fig. 9(a), S6 and Table 2). All formulations containing 2.5 wt% REAs exhibited a single *T*_g_ value close to that of DGEBA_IL40 (139 °C) (Fig. 9(b)) indicating the formation of a single network type, and the molecular weight between crosslinks (*M*_c_) remained comparable (Table S1). In this case, however, the comparable *M*_c_ values suggest that changes in cross-link density are not the primary cause of the reduction in the Young's modulus. Hence, the addition of 2.5 wt% REAs to DGEBA_IL40 likely reduces Young's modulus by disrupting network packing and introducing network heterogeneities (Fig. S7), consistent with a previous report showing that higher heterogeneity, achieved *via* an increase in the cure temperature for the epoxy/amine system, leads to reduction in Young's modulus.^70^

**Fig. 9:**
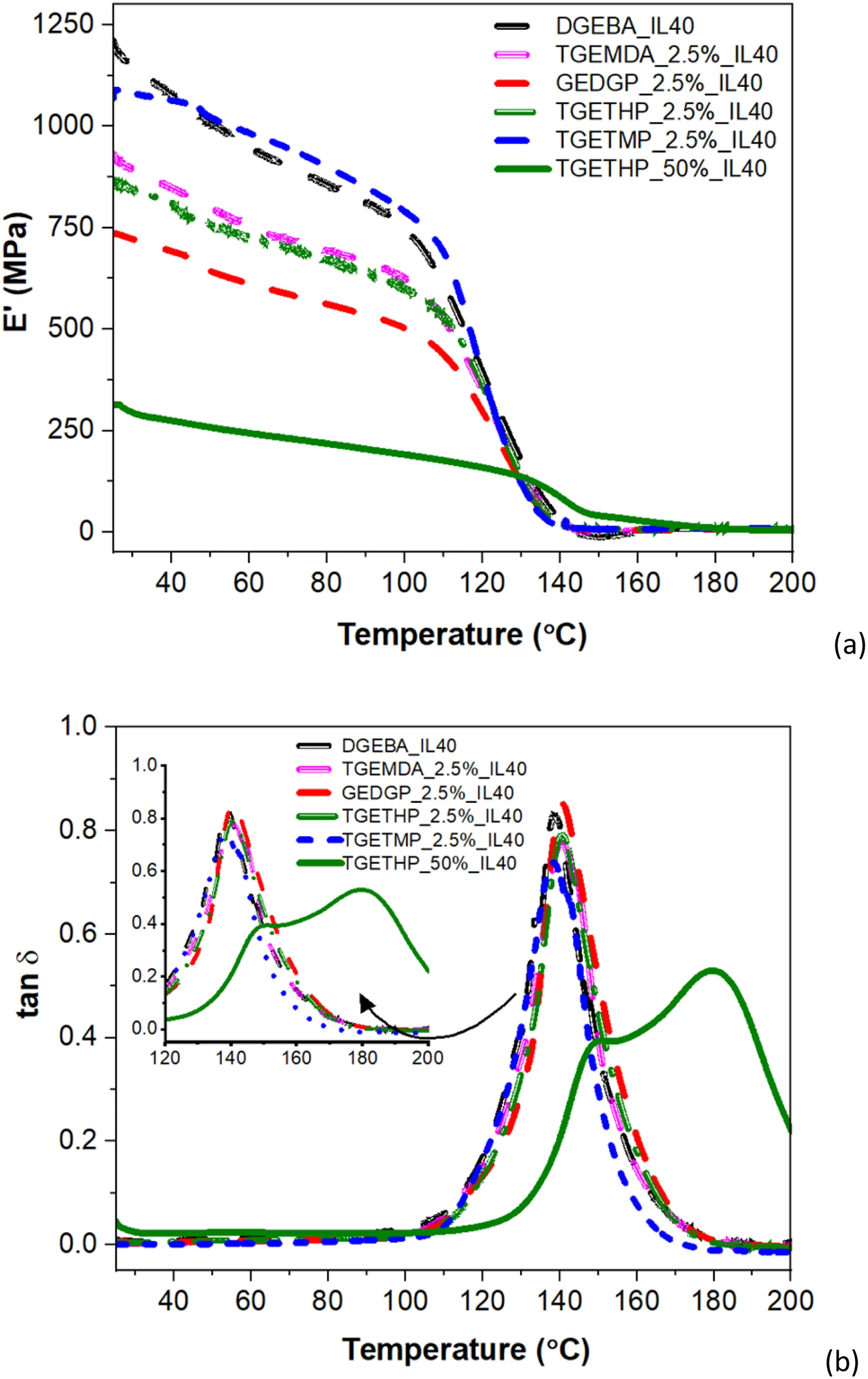
Effect of the composition on the storage modulus (*E*′) (a) and tan *δ* curves (b) for DGEBA-based SEs with 40 vol% EMIM-TFSI.

Moreover, the formulation containing 50 wt% TGETHP displayed markedly different properties with a significant drop in strut sizes and Young's modulus in comparison to those of TGETHP_2.5%_IL40. The pronounced reduction in strut size is likely a contributor to the reduction in Young's modulus, although the higher content of this sterically hindered tri-epoxy additive likely increased the level of network heterogeneity and increased *M*_c_ (Table 2), contributing to the reduction in Young's modulus.^70^

For TGETHP_50%_IL40, DMTA revealed two distinct *T*_g_ values (*ca.* 150 °C and *ca.* 180 °C), indicative of a compositionally heterogeneous network, likely due to differences in the reaction rates of both resins,^73^ resulting in DGEBA-rich and TGETHP-rich regions.^71,72^ Samples synthesised using DGEBA and 50 wt% TGETHP without EMIM-TFSI equally showed two distinct *T*_g_ peaks (Fig. S8) on the tan *δ* curve (*ca.* 160 °C and *ca.* 190 °C). The difference in *T*_g_ values between samples prepared with and without the ionic liquid can be explained by the plasticisation of both networks with EMIM-TFSI.^70,74^ This indicates that the high TGETHP loading promotes a more heterogeneous network than the lower REA loadings.

These findings demonstrate that REAs influence morphology, ionic conductivity and mechanical performance of the DGEBA/EMIM-TFSI RIPS system. The observed ionic conductivity trends indicate that both microscale morphological changes and polarity are affecting ionic transport, whereas the modulus is primarily governed by strut cross-section and network heterogeneity.

### 3.4 Structural electrolyte morphology with CF-CAG reinforcement

In SSCs, the formation of the SE microstructure in the presence of electrode material, especially at the interface, is critical.^55^ Here, we studied the effect of REAs on interfacial morphology by curing the SE formulations in the presence of two CF-CAG sections. The cured sections were then manually delaminated, following a published procedure,^10^ exposing the CF-CAG/SE interface and showing the representative microstructure (Fig. 10).

**Fig. 10:**
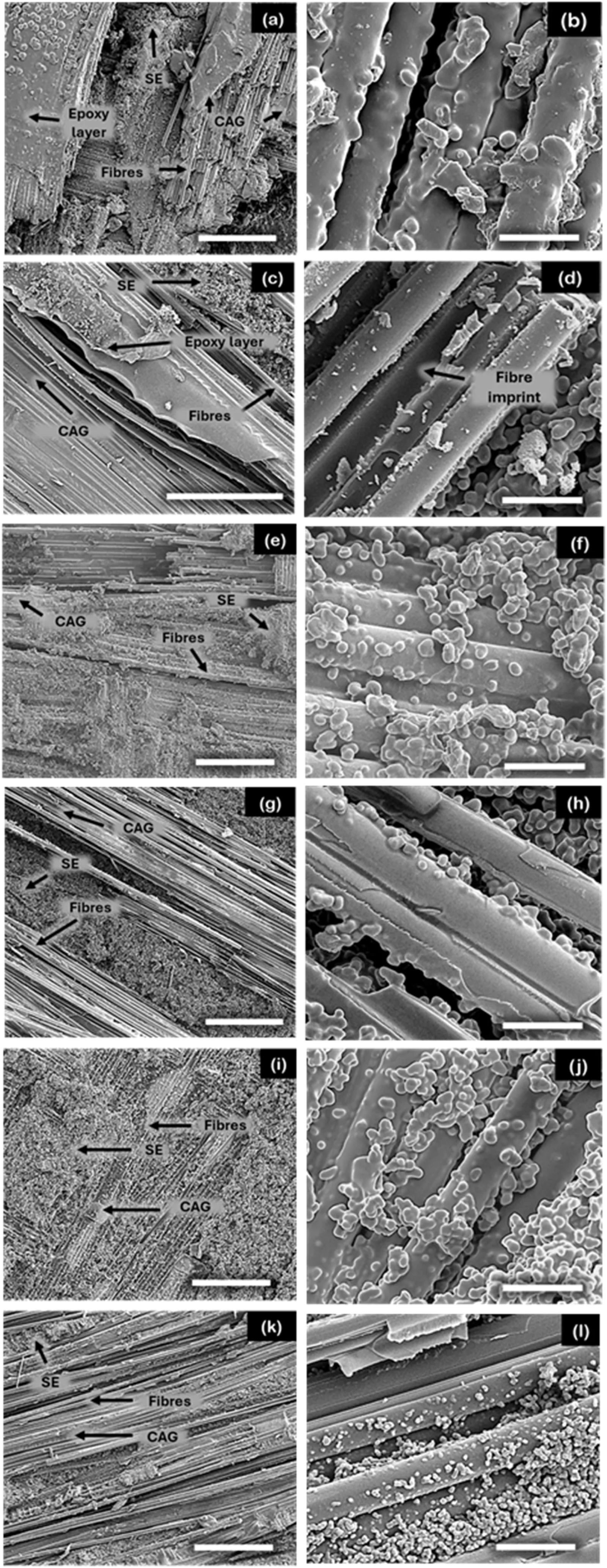
Scanning electron micrographs of the CF-CAG with the DGEBA-based structural electrolytes with 40 vol% EMIM-TFSI: (a and b) DGEBA_IL40; (c and d) TGETMP_2.5%_IL40; (e and f) GEDGP_2.5%_IL40; (g and h) TGEMDA_2.5%_IL40; (i and j) TGETHP_2.5%_IL40; and (k and l) TGETHP_50 wt%_IL40. The scale bar for (a, c, e, g, i and k) is 100 µm and for (b, d, f, h, j and l) is 10 µm.

The bicontinuous phase morphology of DGEBA/EMIM-TFSI structural electrolytes typically exhibited characteristic length scales in the order of microns (Fig. 7), whereas that of the porous network within CF-CAGs is below 100 nm.^11,51^ In the unmodified DGEBA_IL40 system introduction of the CF-CAG led to formation of the microstructure which differed significantly between the polymer close to the fibre surface and the bulk (Fig. 10(a) and (b)). A continuous epoxy sheath with very uneven porosity formed around the fibres. Some regions showed porosity, while others remained smooth with minimal structural features. Delamination disrupted the sheath locally, yet the near-fibre morphology remained distinct, displaying small bead-like structures on the fibres. The sheathing led to cohesive fractures of SE on the fibres, indicating a strong fibre/SE interfacial bond in DGEBA_IL40.^75,76^ A thick epoxy sheath was also observed on the CAG region of CF-CAG, which is undesirable for ion transfer because it may block ionic liquid access to CAG pores, restricting ion transport and reducing electrochemical performance.

As can be seen in Fig. 10(c)–(l), the addition of REAs to DGEBA_IL40 reduced microstructural variation in the SE on the CF-CAG/SE interface. Despite this improvement, fibre and CAG sheathing persisted for the CF-CAG composites prepared with GEDGP_2.5%_IL40 (Fig. 10(e) and (f)) and TGETHP_2.5%_IL40 (Fig. 10(i) and (j)), indicating limited modification of the interfacial morphology relative to DGEBA_IL40. For TGETMP_2.5%_IL40 (Fig. 10(c) and (d)) and TGEMDA_2.5%_IL40 (Fig. 10(g) and (h)) sheathing on fibres and CAG was reduced compared to DGEBA_IL40, but the fracture was less cohesive indicating weaker fibre/SE interfacial bonding. In contrast, when the TGETHP content was increased to 50 wt% (TGETHP_50 wt%_IL40) (Fig. 10(k) and (l), fibre and CAG sheathing was significantly reduced compared to TGETHP_2.5%_IL40, but the delamination was purely interfacial, signifying a reduction in fibre/SE interfacial strength compared to the composite based on DGEBA_IL40.^76^ In samples containing TGETHP_50 wt%_IL40, microstructural variation at the CF-CAG/SE interface was minimal compared to the other formulations, although it remained distinct from that of the bulk.

Bead formation was observed in all additive-containing formulations (Fig. 10(c)–(l)), which is likely associated with locally accelerated curing and enhanced interfacial reactivity.^77^

Consequently, the observed variations in interfacial morphology among the CF-CAG composites and REA systems are likely governed by differences in additive reactivity, their influence on matrix rheology, and the resulting interfacial curing dynamics.^78,79^ These factors should be considered in any future SE formulation development aimed at balancing CF-CAG/SE interfacial behaviour, ionic transport and mechanical performance.

## 4 Conclusions

This study investigated the effects of multi-oxirane ring epoxy additives on the microstructure, mechanical properties, ionic conductivity, and interfacial morphology of DGEBA-based SEs. The introduction of REAs provides a route to reducing the microstructural feature size and changing the rate of phase separation. Low additive loadings (2.5 wt%) had a positive impact on ionic conductivity without compromising the microstructure, whereas high TGETHP content (50 wt%) induced significant microstructural refinement, evidenced by reduced strut size. However, high loading also introduced elevated network heterogeneity, manifested as dual glass transitions and a reduced modulus. Small-angle neutron scattering analysis indicated that a loading of 2.5 wt% TGETMP delayed the onset of phase separation in DGEBA_IL40, demonstrating that REAs can affect curing kinetics and network development. When incorporated into CF-CAG reinforced systems, REAs reduced local microstructural variation at the CF-CAG/SE interface compared to unmodified DGEBA_IL40. Overall, the results indicate that higher-functionality REAs can promote early network formation during curing, influencing both network heterogeneity and phase segregation in bulk and reinforced systems. High network functionality may also increase the polarity of the structural polymer, promote ion dissociation, and thereby enhance ionic conductivity. Among the formulations studied, TGETMP_2.5%_IL40 offered the most balanced overall performance, combining improved ionic conductivity with the smallest reduction in modulus among the bulk SEs and a more uniform CF-CAG/SE interfacial morphology. Overall, this work highlights the strong dependence of DGEBA-based SEs on epoxy additive functionality and ring density, which govern both bulk network formation and interfacial curing behaviour. As a future direction, further modification of the oxirane groups in REAs to cyclic carbonates could provide a route to simultaneously improve ionic conductivity and mechanical properties.

## Conflicts of interest

The authors declare no conflict of interest.

## Supplementary Material

TA-014-D6TA02802K-s001

## Data Availability

The data supporting this article have been included as part of the supplementary information (SI). Supplementary information: SEM micrographs, ionic conductivity measurements, mechanical testing data, and dynamic mechanical analysis data. See DOI: https://doi.org/10.1039/d6ta02802k. The raw SEM data supporting this article are available in the Durham University Research Data Repository and can be accessed at http://doi.org/10.15128/r1707957791. SANS data for formulations discussed in this manuscript is available at https://doi.org/10.5286/ISIS.E.RB2220051; https://doi.org/10.5286/ISIS.E.RB2320239.
