# Supplementary material for: Multi-oxirane additives as property modifiers for DGEBA-based structural electrolytes
Source: J Mater Chem A Mater. 2026 Oct 2;14(63):43850–61. doi: 10.1039/d6ta02802k (PMC13632593; doi:10.1039/d6ta02802k)
Supplement: Supplementary file 1 [file TA-014-D6TA02802K-s001.pdf]

Supplementary Information (SI) for Journal of Materials Chemistry A.
This journal is © The Royal Society of Chemistry

Multi-oxirane additives as properties modifiers for DGEBA based structural electrolytes

Rajat Sharma,[a] Sang Nguyen,[b] Odysseas Kakarelidis,[b] Emile S Greenhalgh,[b] Milo S P Shaffer,[c] Valeria Arrighi,[d] Leide Cavalcanti [e] and Natasha Shirshova *[a]

[a.] Department of Engineering, Durham University, South Road, Durham DH1 3LE, United Kingdom.

[b.] Department of Aeronautics, Imperial College London, South Kensington, London SW7 2AZ, United Kingdom.

[c.] Department of Materials, Imperial College London, South Kensington, London SW7 2AZ, United Kingdom.

[d.] School of Engineering and Physical Sciences, Institute of Chemical Sciences, Heriot-Watt University, Edinburgh EH14 4AS, United Kingdom.

[e.] Science Technology Facilities Council, Isis Neutron and Muon Source, Harwell Campus, Oxfordshire OX11 0QX, United Kingdom.

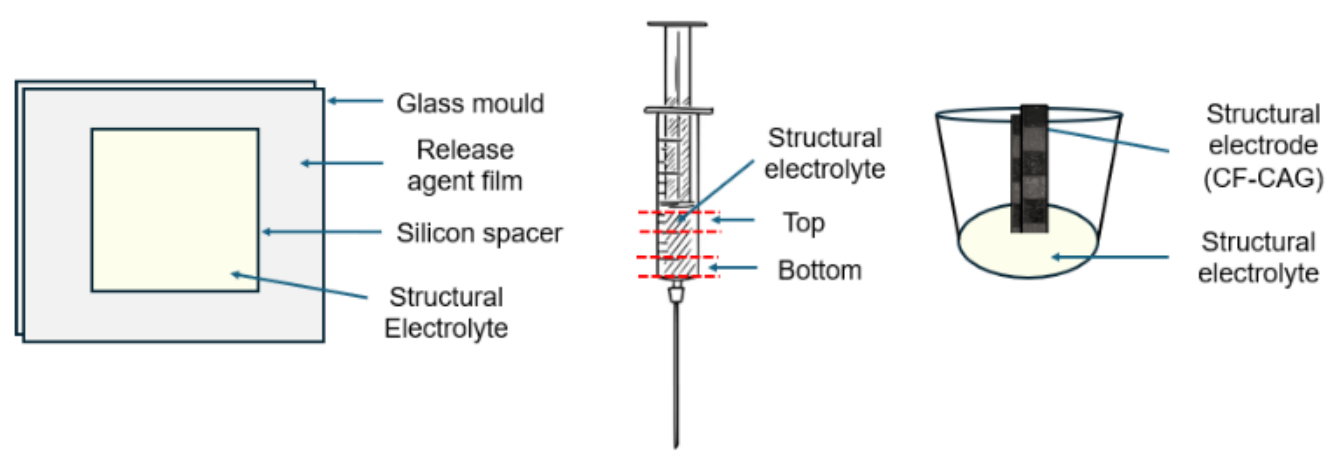

(a) (b) (c)

Figure S1. Sample preparation of structural electrolyte formulations: (a) glass mould for three-point bending tests, (b) syringe for ionic conductivity measurements, and (c) polypropylene beaker with 2 CF-CAG sections.

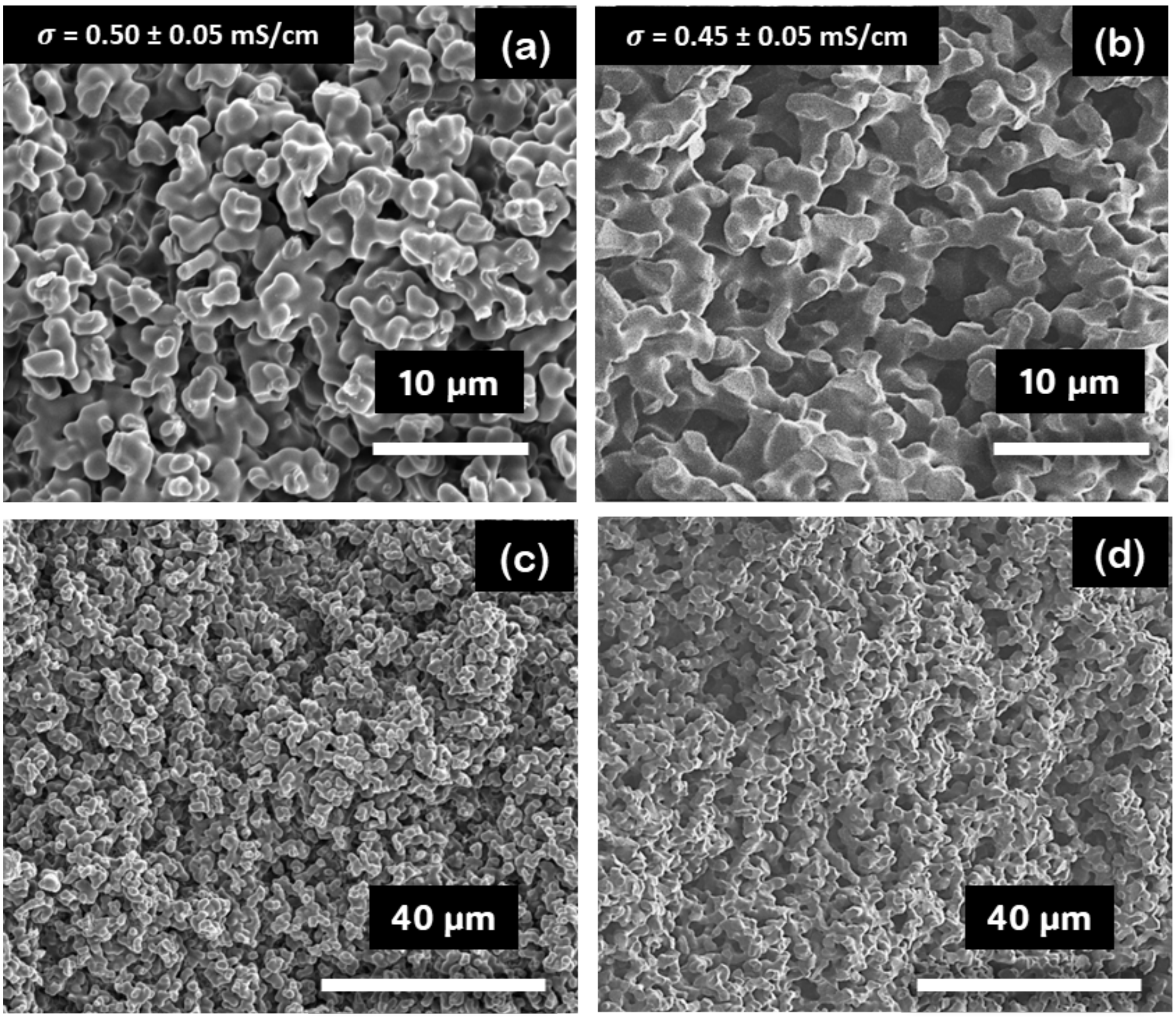

Figure S2. SEM micrographs and ionic conductivity (σ) of DGEBA based SE with 40vol% IL (DGEBA_IL40), cured in (a, c) glass mould, and (b, d) syringe.

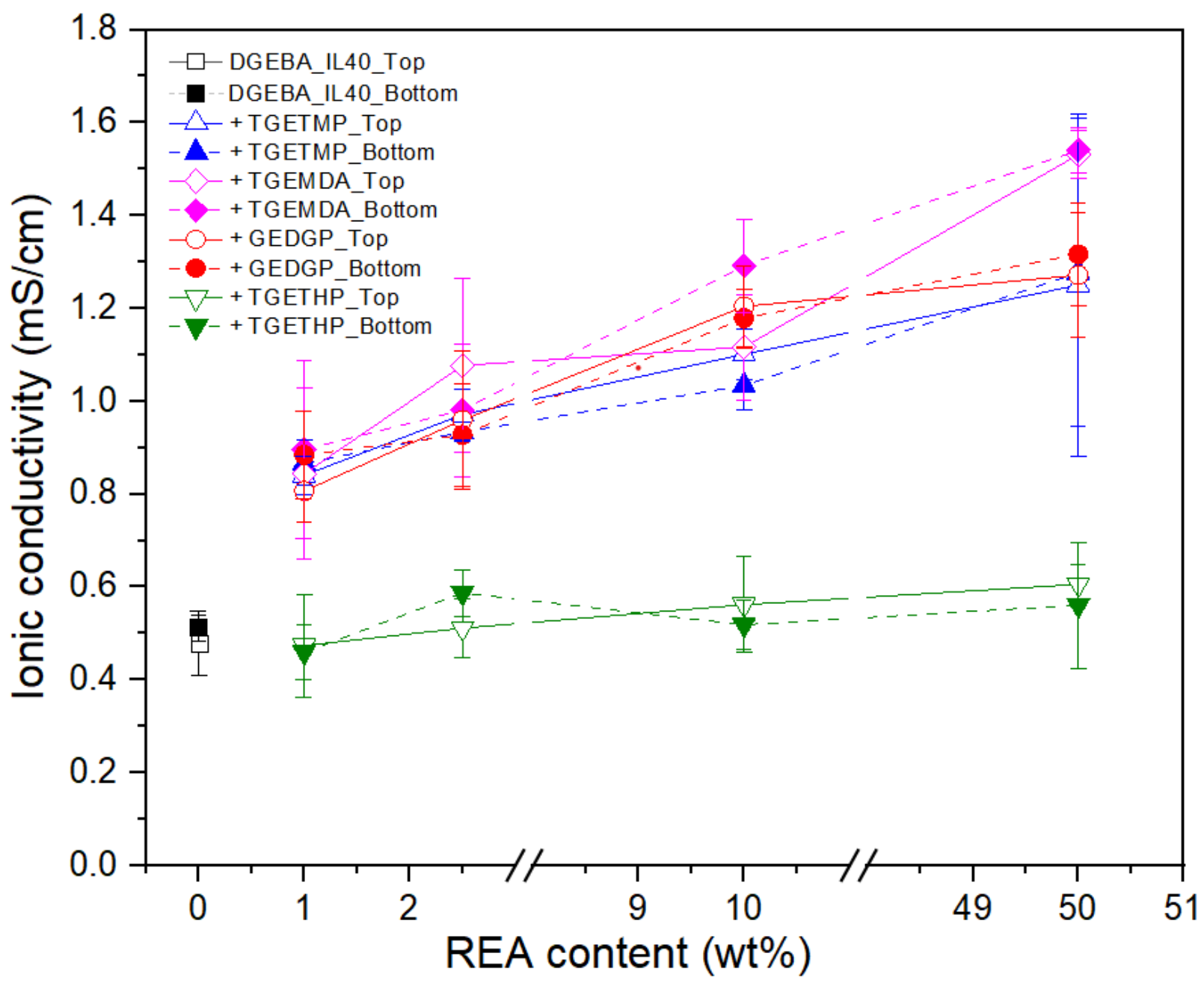

Figure S3. Ionic conductivity at the top and bottom of cylindrical specimens with varying additive concentrations.

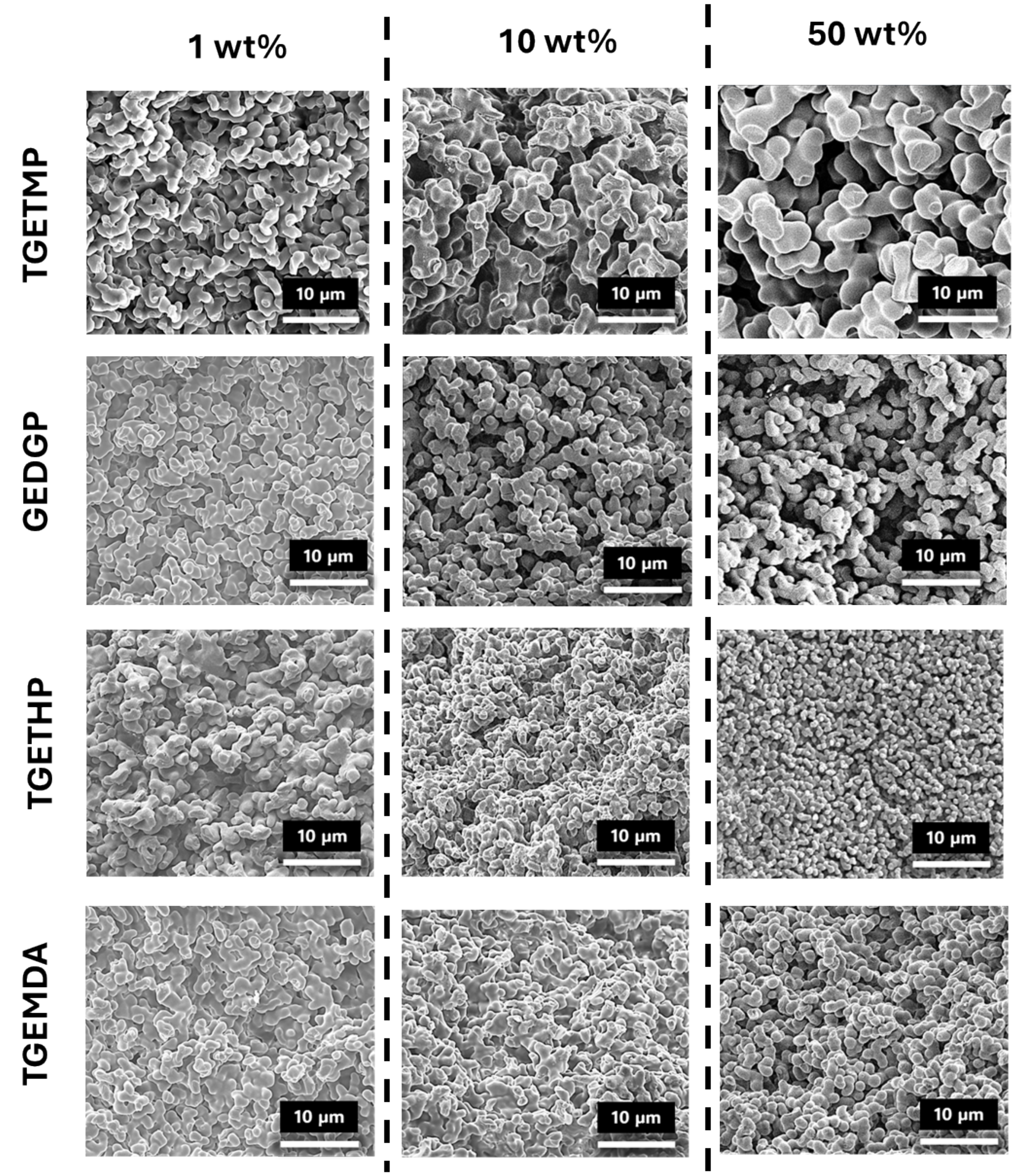

Figure S4. SEM micrographs of DGEBA-based structural electrolytes with 1, 10 and 50 wt% of REA.

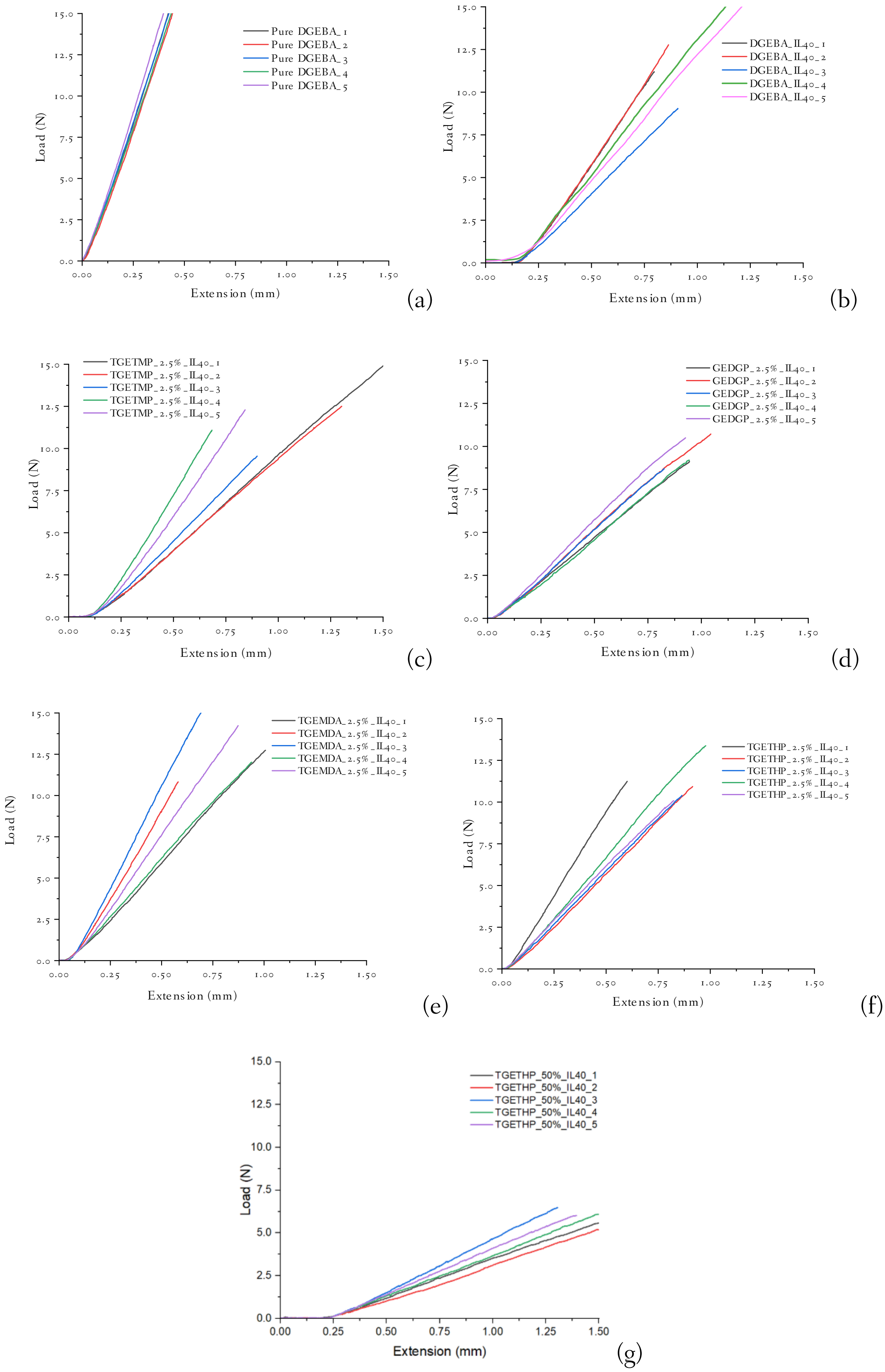

Figure S5. Force–displacement curves from 3 PB tests for DGEBA-based structural electrolytes: (a) pure DGEBA, (b) DGEBA_IL40, (c) TGETMP_2.5%_IL40, (d) GEDGP_2.5%_IL40, (e) TGEMDA_2.5%_IL40, (f) TGETHP_2.5%_IL40, and (g) TGETHP_50%_IL40.

Table S1. Dynamic mechanical properties from DMA measurements of the evaluated structural electrolyte formulations

**Sample code**	**Density (g/cm^3)**	**T_g (° C)**	**tan δ**	**E' at 25 °C (GPa)**	**E' at T_g + 40 °C (MPa)**	**M_c (g/mol)**	**v_e ($mmol/cm^3$)**
**Pure DGEBA**	1.1376 ± 0.0027	160	1.24	2.80	27	500	2.30
**DGEBA_IL40**	1.3053 ± 0.0013	139	0.83	1.20	8	1840	0.71
**TGETMP_2.5%_IL40**	1.3008 ± 0.0012	138	0.77	1.08	8	1830	0.71
**TGEMDA_2.5%_IL40**	1.3206 ± 0.0045	140	0.80	0.85	8	1870	0.71
**GEDGP_2.5%_IL40**	1.3140 ± 0.0056	139	0.73	0.92	8	1850	0.71
**TGETHP_2.5%_IL40**	1.3159 ± 0.0042	140	0.84	0.75	8	1860	0.71
**TGETHP_50%_IL40***	1.3663 ± 0.0047	150/180	0.39	0.31	5	3000/3200	0.43

*Two T_gs were observed for TGETHP_50%_IL40.

E' - Storage modulus; M_c - molecular weight between crosslinks; v_e - cross linking density

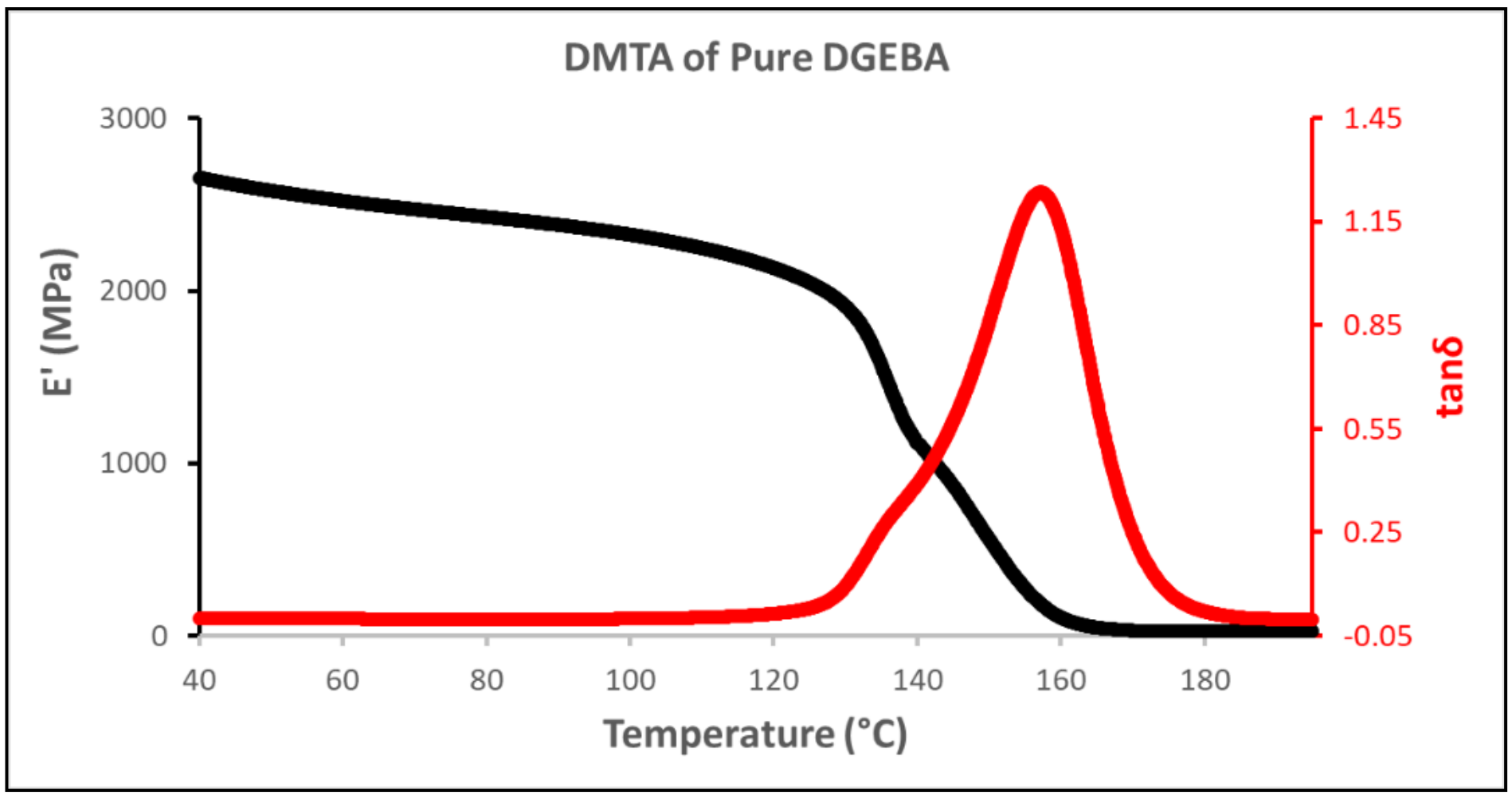

Figure S6. DMA storage modulus (E') and tangent delta (tan δ) curves for pure DGEBA.

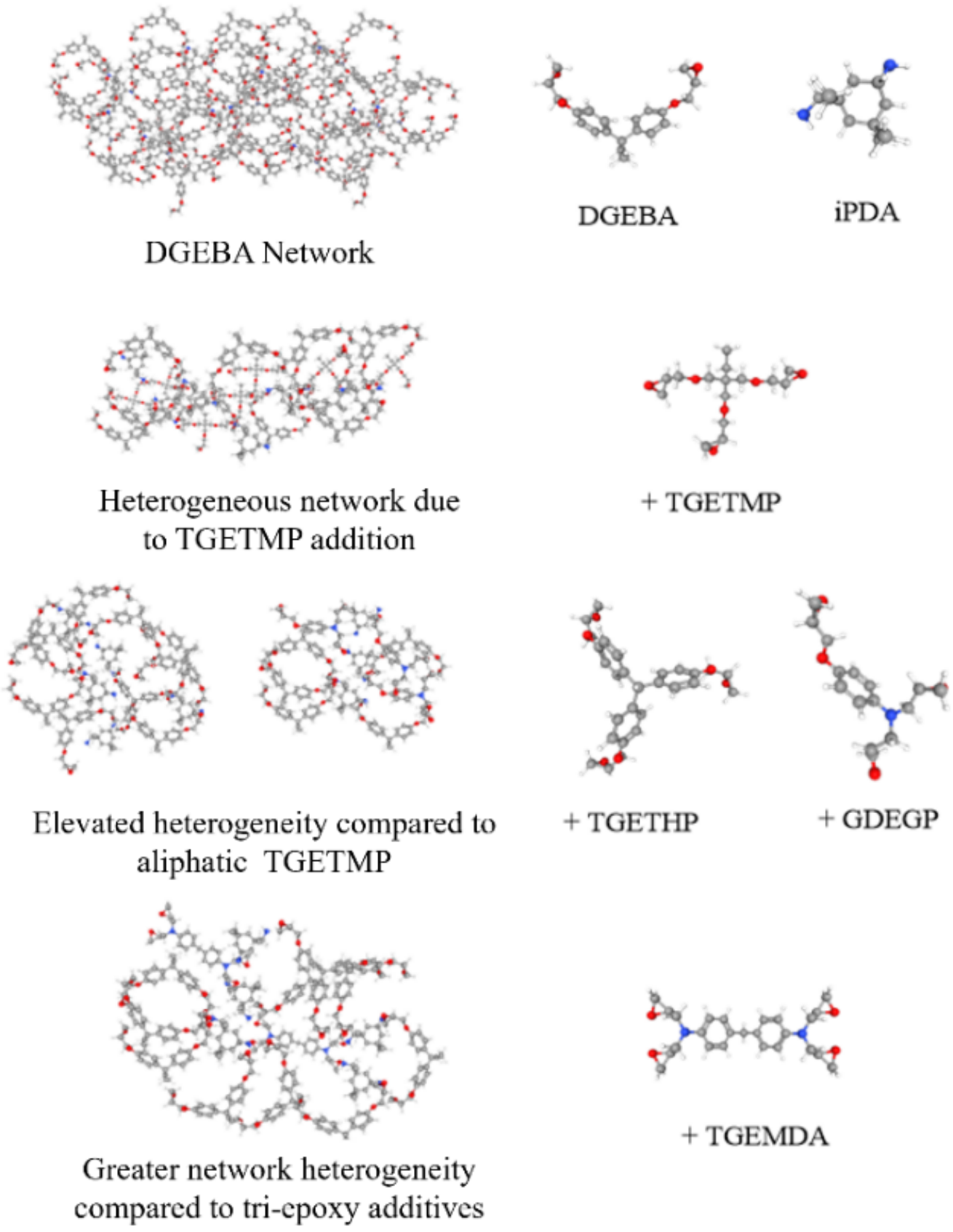

Figure S7. Schematic illustration on the proposed network heterogeneity induced by reactive epoxy additives in the DGEBA/EMIM-TFSI network. Nitrogen and oxygen groups are represented by colour blue and red, respectively.

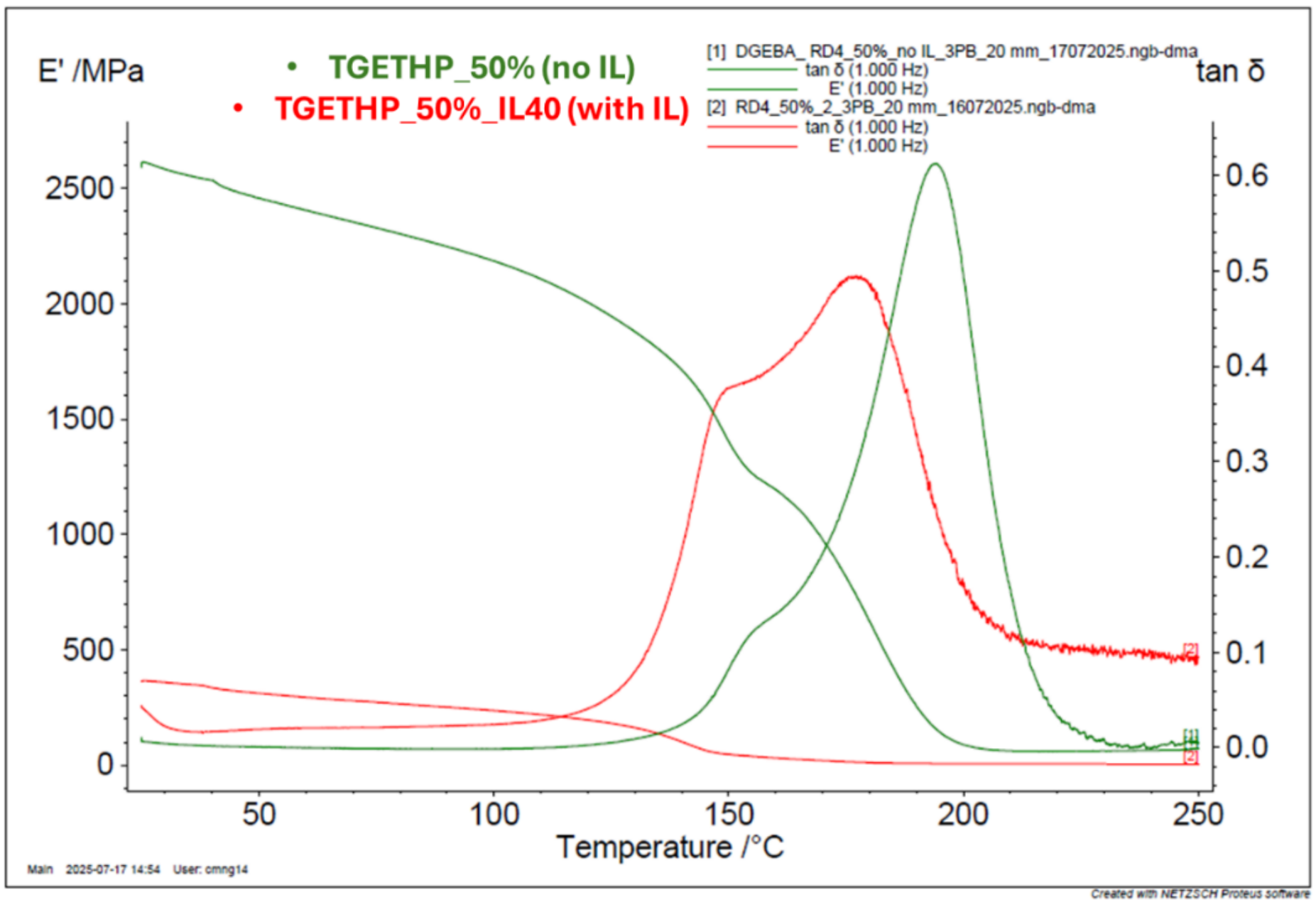

Figure S8. DMA storage modulus (E') and tangent delta (tan δ) curves for TGETHP_50%_IL40 with and without IL.
